# Enzyme assays with supramolecular chemosensors – the label-free approach

**DOI:** 10.1039/d1ra08617k

**Published:** 2022-04-06

**Authors:** Mohamed Nilam, Andreas Hennig

**Affiliations:** Department of Biology/Chemistry, Center for Cellular Nanoanalytics (CellNanOs), Universität Osnabrück Barbarastr. 7 D-49076 Osnabrück Germany andreas.hennig@uni-osnabrueck.de

## Abstract

Enzyme activity measurements are essential for many research areas, *e.g.*, for the identification of inhibitors in drug discovery, in bioengineering of enzyme mutants for biotechnological applications, or in bioanalytical chemistry as parts of biosensors. In particular in high-throughput screening (HTS), sensitive optical detection is most preferred and numerous absorption and fluorescence spectroscopy-based enzyme assays have been developed, which most frequently require time-consuming fluorescent labelling that may interfere with biological recognition. The use of supramolecular chemosensors, which can specifically signal analytes with fluorescence-based read-out methods, affords an attractive and label-free alternative to more established enzyme assays. We provide herein a comprehensive review that summarizes the current state-of-the-art of supramolecular enzyme assays ranging from early examples with covalent chemosensors to the most recent applications of supramolecular tandem enzyme assays, which utilize common and often commercially available combinations of macrocyclic host molecules (*e.g.* cyclodextrins, calixarenes, and cucurbiturils) and fluorescent dyes as self-assembled reporter pairs for assaying enzyme activity.

## Abstract 2

This review comprehensively summarizes the state of the art in label-free supramolecular tandem enzyme assays using supramolecular chemosensors and includes prospective applications in inhibitor screening, optical sensor arrays, and MRI biosensing.

## Graphical abstract

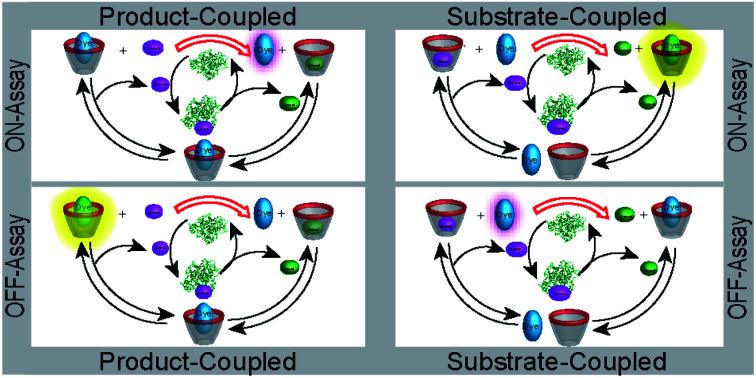

## 1. Introduction

The determination of enzyme activity is a cornerstone in numerous research and application areas. For example, enzyme assays are widely used in clinical diagnosis, drug development, environmental pollution detection, and chemical biological research.^1–3^ Optical spectroscopic methods and, in particular, fluorescence-based methods are by far the most popular, because they are more sustainable than radioactive assays, enable a rapid and highly sensitive signal read-out within a short time, and allow continuous monitoring.^4^ Moreover, fluorescence-based assays can be easily miniaturized, which is especially useful for drug discovery by high-throughput screening (HTS) in the pharmaceutical industry, where large libraries of synthetic compounds are screened for their potential to serve as a drug lead structure against a specific biological target.^5–10^

**Fig. 1:**
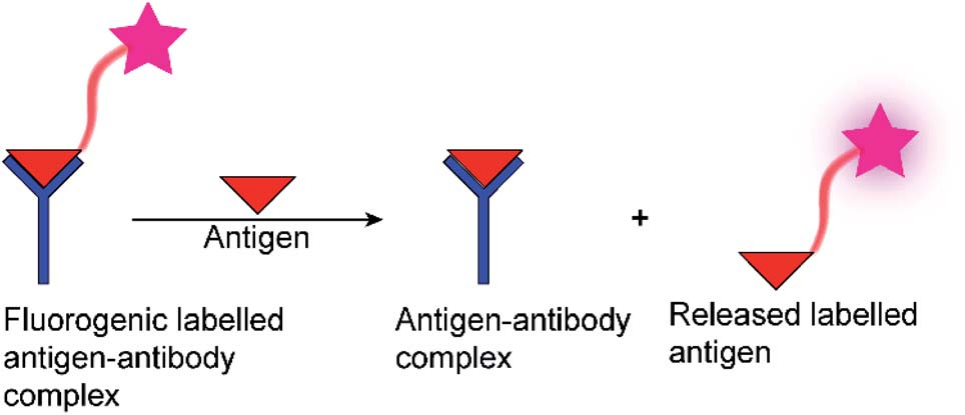
Schematic diagram of a fluorescence immunoassay.

Often, fluorogenic substrates are used, which generate a fluorescence response, for example, after cleavage by proteases or other hydrolases.^11–17^ For other types of enzymes, fluoroimmunoassays with fluorescently labelled antigens and antibodies are used (Fig. 1), in which a fluorescently labelled antigen is displaced from an antibody binding site by the label-free product of an enzymatic reaction (Fig. 1).^2,18^ Fluorescence assays are thus an established technology for high-throughput screening application (HTS),^19^ in drug discovery, clinical diagnostics,^20,21^ and cancer-biomarker.^22,23^ Nonetheless, these approaches have also certain disadvantages. Fluoroimmunoassays comprise often heterogeneous assays with several incubation and washing steps and the exchange kinetics of antibodies are often too slow to allow continuous monitoring, even in a homogenous assay format. Moreover, fluorogenic substrates contain large aromatic dyes, which can interfere with antigen–antibody binding or with the binding of the substrate to the enzyme active site.^2,12,15^

It has been shown in the last fifteen years, that artificial, supramolecular receptors can complement existing enzyme assay technologies. Such supramolecular approaches to enzyme assays rely on supramolecular chemosensors, which are capable to transform chemical information into useful signals and which have found wide-spread use for the detection of biomolecules.^24,25^ The main assets of supramolecular chemosensors include the negligible large-scale production costs compared to antibodies, a much faster exchange kinetics compared to antibodies, and that they offer complementary molecular recognition principles. For example, supramolecular host molecules are designed to bind small, low-molecular weight guests, whereas it is usually rather difficult to raise specific antibodies against small, abundant biomolecules such as amino acids. In addition, supramolecular chemosensors can benefit – counterintuitively – from a lower selectivity compared to antibodies, because this allows to monitor various related enzymatic transformation with the same chemosensor; typically, a supramolecular chemosensor that discriminates substrate and product is sufficient.^26^ Supramolecular approaches to enzyme assays present nowadays an attractive alternative to established enzyme assays and enable a label-free, continuous fluorescence monitoring of an increasing number of enzymatic reactions.

The aim of this review is to summarize the current state-of-the art in supramolecular approaches to enzyme assays. We provide an introduction to initial efforts from the supramolecular chemistry community to combine supramolecular chemosensing principles with the detection of enzymatic activity (Sections 2 and 3) and then summarize the foundations of “supramolecular tandem enzyme assays”, which involve the use of fluorescent supramolecular host–dye reporter pairs in detecting enzymatic conversions of small substrates (Section 4) and peptides and proteins (Section 5). In the final part of the review (Section 6), we present potential applications of supramolecular enzyme assays, which include, for example, high-throughput screening (HTS) drug discovery, clinical diagnostics, and molecular imaging.

## 2. Enzyme assays with synthetic chemosensors

**Fig. 2:**
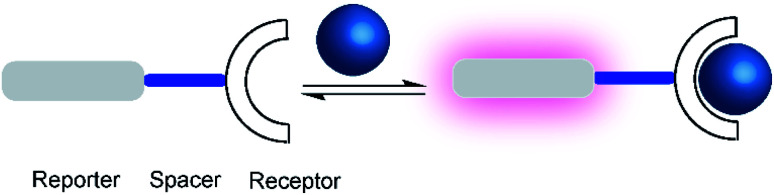
Schematic representations of a supramolecular chemosensor based on the receptor–spacer–reporter design. A binding unit, which can modulate the fluorescence spectroscopic properties of the reporter dye is covalently connected by a spacer. Binding of an analyte (blue) to the receptor affects the photophysical properties, for example, leading to a fluorescence enhancement (purple).

One of the first fluorescent chemosensors was developed in 1867 by F. Goppelsröder to detect Al^3+^, which forms a strongly fluorescent chelate with morin.^27^ This prompted the subsequent development of innumerable different fluorescent chemosensors. Supramolecular chemosensors are commonly based on host–guest complexation, in which the analytes reversibly bind with the chemosensors. An elegant and very versatile approach to supramolecular chemosensing is the “receptor–spacer–reporter” approach (Fig. 2), which was initially developed by de Silva and co-workers and could be successfully applied to detect cations, anions, small neutral molecules, and biomacromolecules.^28^

The “receptor–spacer–reporter” design is based on three components: a receptor site that reversibly binds a particular analyte, a chromophore or fluorophore that serves as a signalling unit, and a spacer, which connects the receptor and reporter within the same compound. For example, an aza-crown ether can be connected to the fluorophore, which is able to alter the optical properties of the fluorophore by quenching the fluorescence through photoinduced electron transfer (PET).^29^ Binding of the analyte, *e.g.* a sodium cation, changes the frontier orbital energy levels of the aza-crown ether and thereby prevents quenching by PET; this leads to a turn-ON fluorescence response of the chemosensor. This system was further extended to other metal ions such as potassium and calcium.^29–31^ Nowadays, it is integrated into point-of-care devices used to assess the electrolyte concentration in blood serum samples in both humans and animals.^32^

**Fig. 3:**
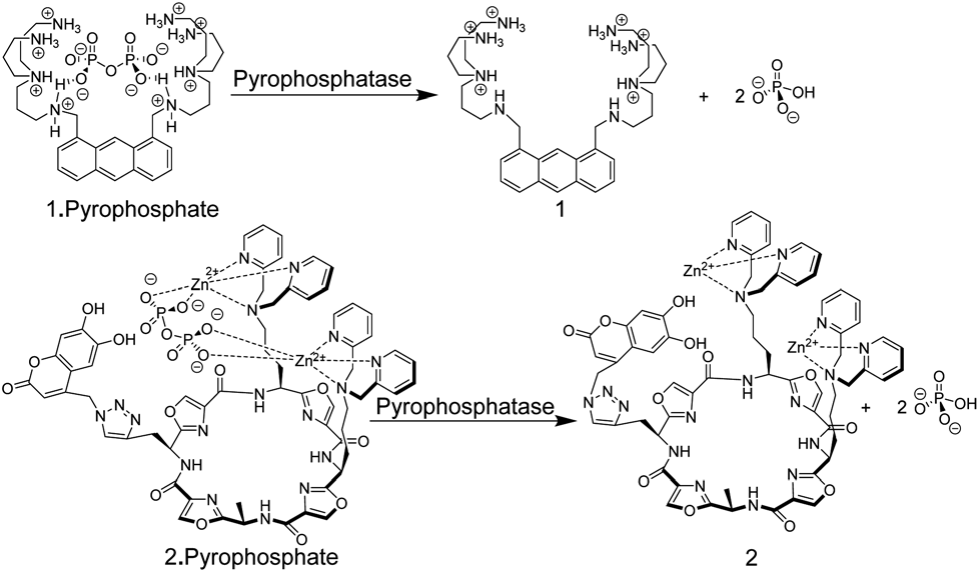
Pyrophosphate chemosensors for the detection of pyrophosphatase activity leading to hydrolysis of pyrophosphate into monophosphate.

Chemosensors based on the receptor–spacer–reporter approach have been also early on explored for assaying enzyme activity. Czarnik and co-workers synthesized the anthracene-based chemosensor 1 containing polyammonium groups as receptor unit, which allows cooperative chelation and fluorescence detection of pyrophosphate but not monophosphate.^33^ This selectivity was applied to monitor the hydrolysis of pyrophosphate into monophosphate by the enzyme pyrophosphatase (Fig. 3).

More recently, an alternative chemosensor to monitor pyrophosphatase activity was reported by Jolliffe and co-workers, who attached two Zn^2+^–dipicolylamino (DPA) units as a recognition motif and a coumarin dye as a reporter unit to a cyclic peptide scaffold to afford chemosensor 2.^34^ The chemosensor showed a high selectivity and affinity for pyrophosphate. In its unbound state, 2 showed a comparably low fluorescence intensity (potentially due to intramolecular association of the catechol unit in the coumarin with the Zn^2+^–DPA units), whereas addition of pyrophosphate led to an increase in fluorescence intensity, implying intramolecular displacement of coumarin from Zn^2+^–DPA units upon pyrophosphate binding. The potential application of 2 in an enzyme assay was demonstrated through monitoring of pyrophosphatase activity. Introduction of pyrophosphatase into the reaction mixture containing 2 and pyrophosphate, resulted in hydrolysis of pyrophosphate into monophosphate. The latter binds much more weakly to 2 such that coumarin can again bind with Zn^2+^–DPA units of 2. This afforded a decrease in fluorescence intensity in response to pyrophosphatase activity.

**Fig. 4:**
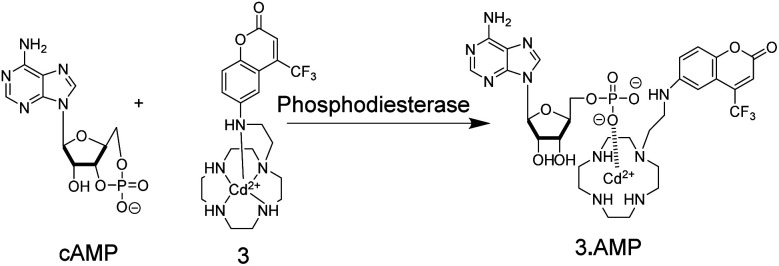
Detection of phosphodiesterase activity by chemosensor 3. The substrate cAMP does not bind to the Cd^2+^-cyclen unit, whereas the product AMP binds strong enough to displace the aminocoumarin dye from its axial position leading to a red-shift in fluorescence intensity.

Supramolecular chemosensors were also used to monitor phosphodiesterase (PDE) activity with nucleosides (Fig. 4).^35^ Kikuchi and co-workers synthesized 3, which includes 7-amino-4-trifluoromethylcoumarin as a reporter fluorophore and Cd^2+^-cyclen(1,4,7,10-tetraazacyclododecane) as an anion receptor unit. This chemosensor displayed micromolar affinity to nucleoside mono-, di-, and triphosphates, whereas cyclic adenosine monophosphate (cAMP) had a much weaker affinity to 3. Thus, the activity of PDE transforming cAMP to AMP can be monitored by receptor 3-Cd^2+^. Interestingly, the displacement of the axial amino group of the coumarin unit by the product AMP from the Cd^2+^-cyclen unit led to a red shift of the fluorescence spectra enabling ratiometric monitoring, which was, however, not demonstrated.

**Fig. 5:**
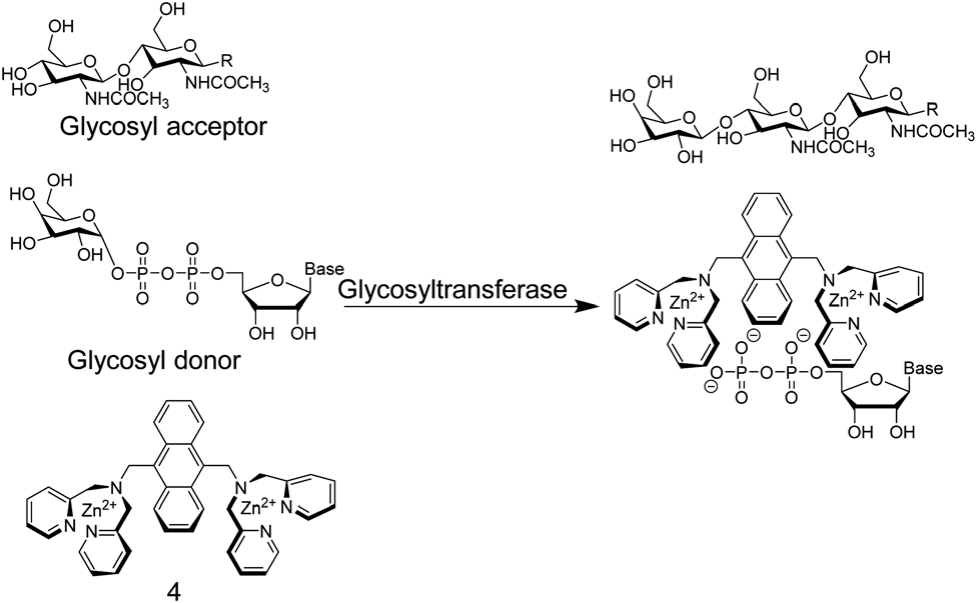
Chemosensor 4 binds nucleotide diphosphates more strongly than the glycosylated pyrophosphate diesters, which enables the monitoring of glycosyltransferase activity.

In another study,^36^ Hamachi and co-workers exploited the phosphate binding properties of Zn^2+^–DPA units to monitor the activity of glycosyltransferases with chemosensor 4 (Fig. 5). 4 binds phosphate monoesters more strongly than phosphate diesters and gives a fluorescence increase upon binding, because PET is less effective in the phosphate complex. Glycosyltransfer could thus be monitored by the strong binding of the reaction product uridine 5′-diphosphate (UDP), after transfer of the glycosyl residue from the glycosyl donor to the glycosyl acceptor. The biocompatibility of chemosensor 4 was confirmed by reproducing literature-known trends of a wide variety of enzyme kinetic parameters. This included combinations of different glycosyltransferases, namely β-1,4-galactosyltransferase, α-1,3-galactosyltransferase, and α-2,3-sialyltransferase in combination with different substrates such as chitobiose, *N*-acetylglucosamine, glucose, *N*-acetyllactosamine, lactose, and galactose.

## 3. Enzyme assays by indicator displacement

In the preceding section, we have summarized methods to monitor enzyme activity using supramolecular receptors, which were covalently modified with a fluorogenic or chromogenic reporter unit. The employed receptor–spacer–reporter strategy has, however, its limitations in the substantial synthetic effort to covalently connect the reporter dye and the receptor unit. An alternative strategy that bypasses such synthetic challenges is the indicator displacement assay (IDA), which was introduced into the supramolecular chemistry community by the group Eric V. Anslyn in the late 1990s to 2000s.^37,38^

IDAs are dependent on indicator dyes that afford significant absorption or fluorescence spectral changes upon complexation with supramolecular receptor molecules. The addition of a competing guest molecule that can displace the dye from the receptor principle is nowadays routine to determine the affinity of supramolecular guest molecules not bearing a chromophoric or fluorophoric unit by competitive titrations,^39–41^ it has been used the elucidation of mechanistic pathways and for material characterizations,^42–44^ and it was first used to detect anion binding to cyclodextrins.^45^ The conceptual advancement behind an IDA is the analytical application, namely that the competitor is an analyte that can to be detected or quantified based on the competition between the receptor/dye and receptor/analyte complex.^46^ A sufficiently strongly binding analyte will displace the indicator at appropriate analyte concentrations resulting in a measurable optical output signal. The IDA-based sensing principle has been applied to a large variety of analytes, *e.g.* citrate, glucose-6-phosphate, tartrate, malate, or nitrate.^47^ The applied detection methods include absorption and fluorescence spectroscopy.

**Fig. 6:**
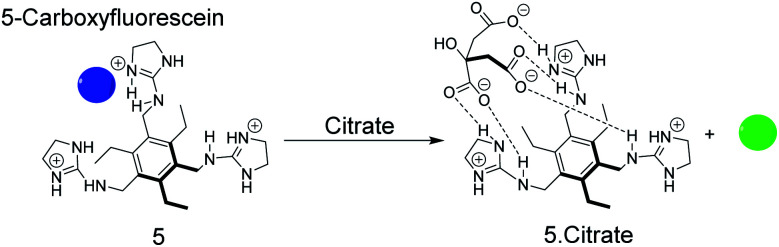
Sensing of citrate by displacement of 5-carboxyfluorescein as the indicator dye from the receptor 5. The dye is quenched in its complexed form (blue) and liberation of the free dye regenerates the fluorescence (green).

As an example, the tripodal guanidinium-based receptor 5 (Fig. 6) has been applied to assess the citrate concentration in various soft drinks by fluorogenic IDA.^47^ The binding affinity between 5 and citrate was undetectably low in water, but in 25% (v/v) 5 mM Hepes, pH 7.4 in methanol, the binding affinity for 5 and citrate was found to be 2.9 × 10^5^ M^−1^. Under these conditions 5-carboxyfluorescein binds with mM molar affinity (*K*_a_ = 4.7 × 10^3^ M^−1^) to 5 leading to fluorescence quenching.^38^ The millimolar concentrations of citrate in soft drinks thus led to displacement of 5-carboxyfluorescein, which was detectable by UV-Vis and fluorescence spectroscopy. Potential interferences of the sensor system include phosphate buffer^47^ and succinate which has also a considerable binding affinity with 5 (*K*_d_ = 4.5 mM).^38,48^

**Fig. 7:**
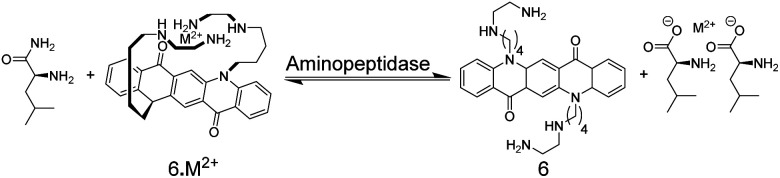
The enzymatic hydrolysis of l-leucinamide by leucine aminopeptidase was followed with chemosensor 6. The reaction product is the α-amino acid l-leucine, which serves as a bidentate ligand for Cu^2+^ and Ni^2+^ and thus displaces M^2+^ from 6. The hydrolysis of *N*-acetyl-l-methionine into l-methionine by acylase I could be similarly followed.

The earliest example of an IDA-based enzyme assay was reported by Reymond and co-worker, which used, quite unconventionally, metal ions as binding motifs for substrate and product discrimination (Fig. 7).^49^ The quinacridone-derived fluorescent dye 6 is strongly quenched by energy transfer to complexed Ni^2+^ and Cu^2+^ metal ions. The affinity of 6 to Ni^2+^ and Cu^2+^ was, however, comparably weak for a typical chelate complex, and bidentate ligands such as α-amino acids could efficiently compete for Ni^2+^ and Cu^2+^ binding with 6 leading to a recovery of the fluorescence of unbound 6. The hydrolases acylase I and leucine aminopeptidase afford the α-amino acids l-methionine and l-leucine by hydrolysis of the substrates *N*-acetyl-l-methionine and l-leucinamide, respectively. Since the latter are incapable of coordinating to Ni^2+^ or Cu^2+^, monitoring of acylase I and leucine aminopeptidase activity should therefore become possible. Unfortunately, continuous monitoring was only shown with significant amounts of organic co-solvents (40% DMSO or 40% DMF), which was required to solubilize 6 and which reduced the activity of the enzymes. The native enzyme activity could, however, be determined in an aliquot assay, in which aliquots from the enzymatic reaction in water were successively withdrawn and diluted with the organic solvent containing water-insoluble 6.

**Fig. 8:**
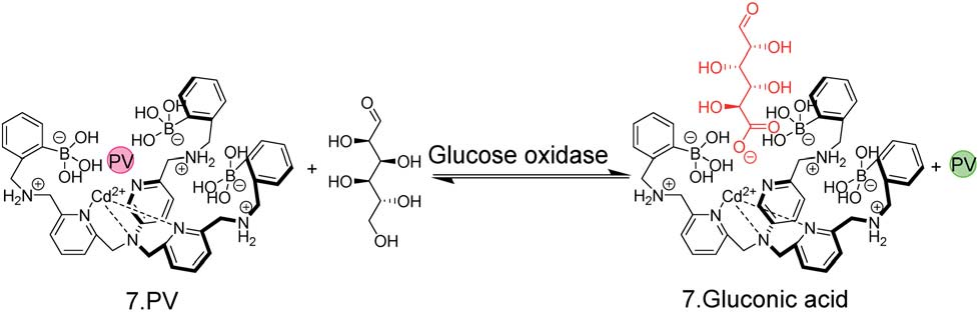
A colorimetric, IDA-based enzyme assay for glucose oxidase. The self-assembled chemosensor 7 binds carboxy and phosphor sugars, but not aldose or ketose carbohydrates. Oxidation of glucose by glucose oxidase generates gluconic acid. Aliquots from the enzyme assay mixture were diluted with MeOH and the enzymatic reaction product displaced the indicator dye pyrocatechol violet (PV) from 7 leading to a colour change from red to yellow.

The possibility to detect enzyme activity with absorption-based IDAs was subsequently also shown by Anslyn and co-workers.^50^ The receptor 7 has a high affinity to gluconic acid, which is the product of the enzymatic oxidation of glucose with glucose oxidase. Pyrocatechol violet (PV) was selected as the indicator dye, which forms a purplish-red coloured boronate ester with 7 (Fig. 8). Also here, continuous monitoring was not possible, because the receptor 7 has insufficient water solubility and required 75% MeOH as an organic co-solvent, but an aliquot assay could be demonstrated, in which 250 μL aliquots were withdrawn from the enzyme assay mixture and diluted with 750 μL MeOH for detection. Within the progress of the enzyme reaction, the detection solution became successively yellowish-green, which is the colour of the unbound dye (Fig. 8).

**Fig. 9:**
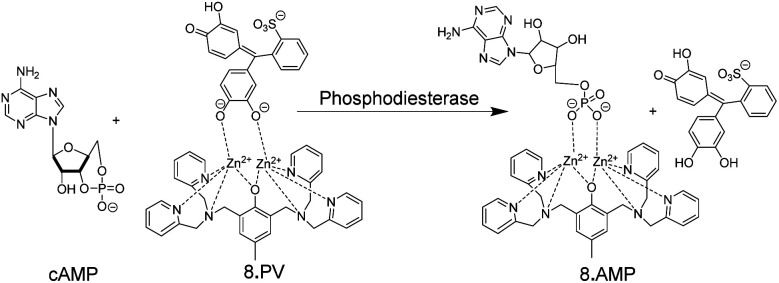
Colorimetric IDA-based phosphodiesterase assay suitable for continuous absorption monitoring. Pyrocatechol violet (PV) as indicator dye is bound by 8. The substrate cAMP does not bind, but the product AMP displaces PV from leading to a colour change.

An early example of an absorption-based, homogeneous assay format with the possibility for continuous monitoring of enzyme activity in solution was developed by Kim and co-workers.^51^ They used the metal ion chelating properties of PV in conjunction with receptor 8 for a PDE assay (Fig. 9). 8 can also bind to the phosphate ester of AMP, but not to the phosphate diester of cAMP. Hydrolysis of cAMP by PDE into AMP continuously displaced the dye pyrocatechol violet generating an absorption change. Compared to commercial HTS assays, which are based on luminescence and can, thus, be easily adapted to microtiter plate, the chemoreceptor-based assay has a much lower sensitivity (<10 μM AMP) due to the absorption-based measurement.

## 4. Supramolecular tandem enzyme assays

### 4.1 Supramolecular host–dye reporter pairs

**Fig. 10:**
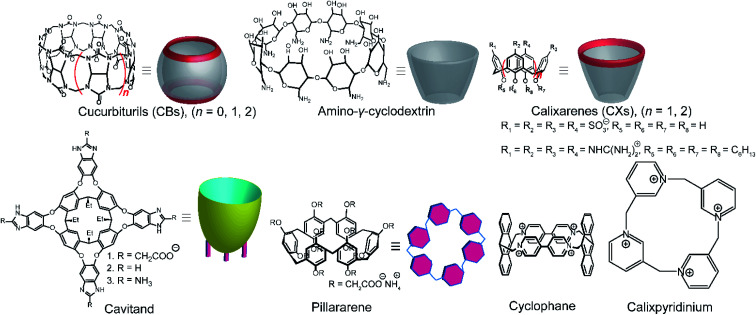
Chemical structures and cartoon representation different macrocycles.

**Fig. 11:**
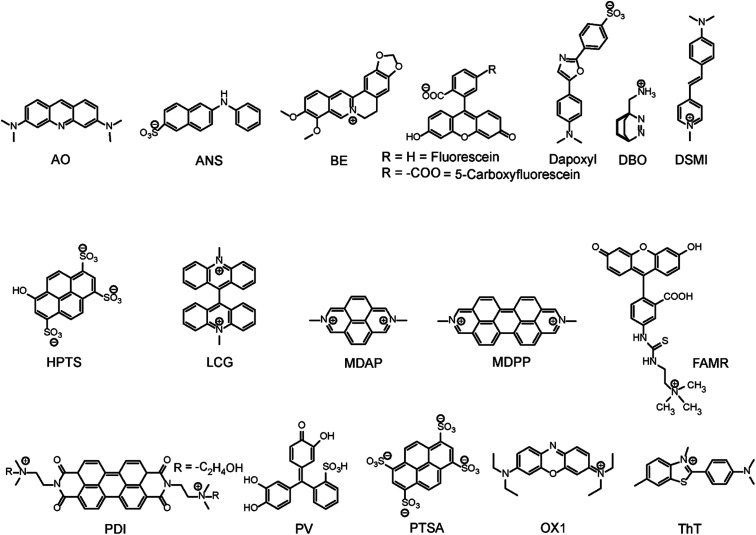
Chemical structures of indicator dyes.

Among various accessible methodologies for monitoring enzymatic activity, fluorescence-based methods are usually preferable due to their sensitivity, short read-out times, and their possibility for continuous monitoring.^4^ Prototypical classes of macrocyclic host molecules such as cucurbiturils, calixarenes, cavitands, cyclodextrins, pillararenes, and cyclophanes (Fig. 10) are well-known to bind with a large variety of fluorescent dyes (Fig. 11) and thereby alter their photophysical properties.^52,53^

**Fig. 12:**
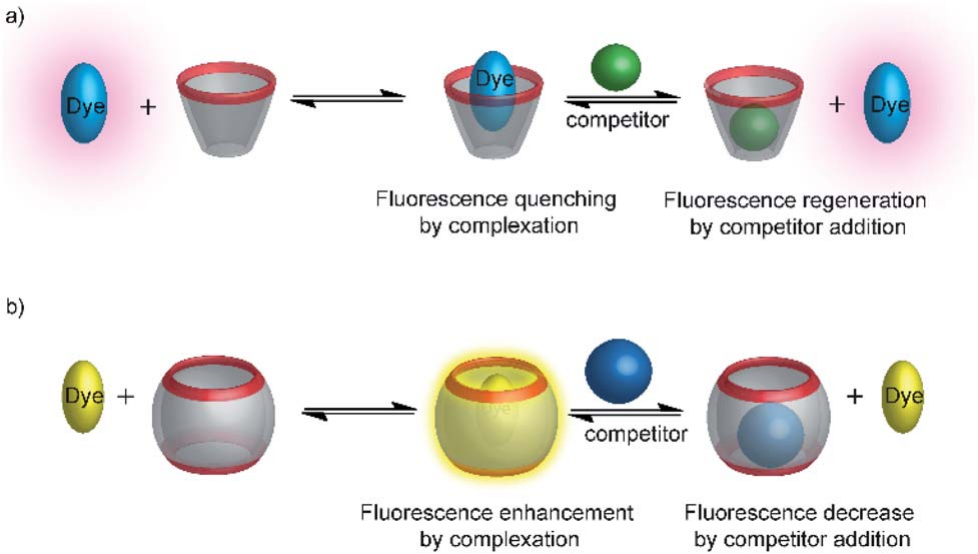
Supramolecular macrocycle-dye complexes afford self-assembled chemosensors (=reporter pairs) for analyte sensing. In an ON-IDA (a), fluorescence quenching is observed upon macrocycle-dye complex formation and analyte addition affords a fluorescence increase; in an OFF-IDA (b) fluorescence enhancement results from macrocycle-dye binding and analyte addition gives a fluorescence decrease.

Typical effects include fluorescence enhancement or quenching (Fig. 12), whereas other effects, such as changes in the fluorescence lifetime and anisotropy have also been noted.^54–56^ As a consequence, a large variety of host/dye complexes have been investigated in large detail with respect to their photophysical properties,^52^ and certain host/dye combinations were also applied as chemosensors in ON- (Fig. 12a) or OFF-IDAs (Fig. 12b).^26^ The best performing combinations of various hosts and dyes, which showed a large fluorescence response and a high affinity, were summarized by Nau and co-workers, who also coined the name “reporter pair”.^26^ The reporter pairs have been used to assess the absolute concentrations of analytes as well as binding affinities to macrocycles.^57^

Another requirement to label a host–dye combination as a reporter pair is a sufficiently fast exchange time. Typical exchange times of fluorescent dyes with hosts are on the microsecond to millisecond time scale.^58^ This is much faster than the reaction rates in typical enzyme assays, which are usually set up to proceed within minutes. Reporter pairs are thus ideally suited to follow dynamic changes in analyte concentration, in particular in response to an enzymatic reaction.

### 4.2 Product-selective supramolecular tandem enzyme assays

**Fig. 13:**
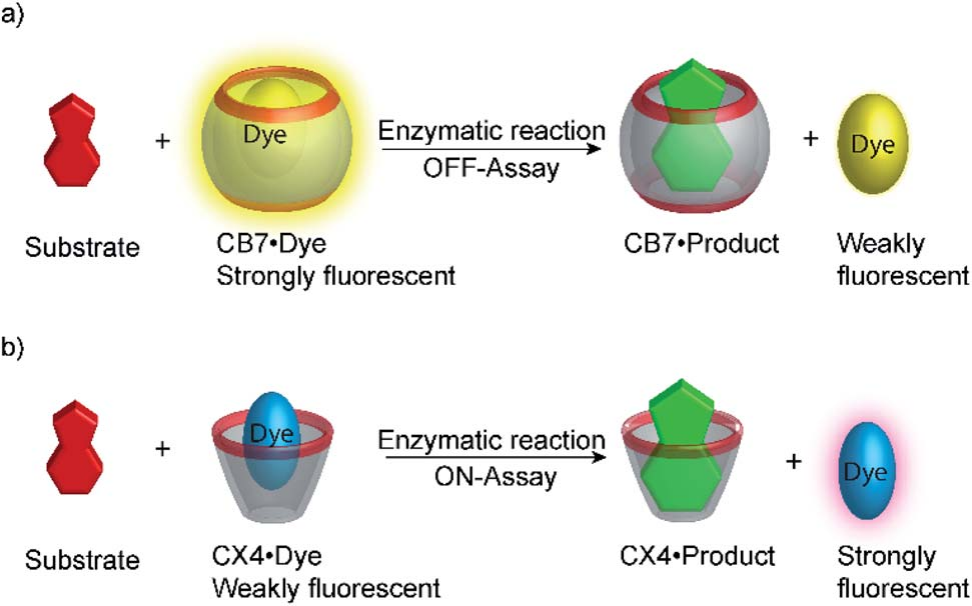
Principle of product-selective supramolecular tandem assays. A weak competitor (substrate, red) is converted into a strong competitor (product, green) by an enzymatic reaction. (a) Switch-OFF variant with a strongly fluorescent reporter pair (*e.g.* CB7/dapoxyl or CB7/BE). (b) Switch-ON variant with a non-fluorescent reporter pair (*e.g.* DBO/CX4 or LCG/CX).

Supramolecular tandem enzyme assays present a broadly applicable approach for monitoring enzyme activity in a facile and accessible manner. The most intuitive way to set up a supramolecular tandem enzyme assay is in its product-selective variant (Fig. 13a).^59^ In a product-selective tandem enzyme assay, the substrate shows negligible affinity to the host and the product shows a very high affinity. The enzymatic reactions thus generate a strong competitor, which displaces the dye from the cavity leading to a pronounced fluorescence response. This principle thus immediately affords a highly sensitive real-time fluorescence monitoring of enzyme activity with label-free substrates.

Product-coupled tandem assays were initially developed for amino acid decarboxylases.^59^ This class of enzymes removes the carboxylic acid group from amino acids leading to an overall increase of the positive charge and were thus monitored with the cation receptors cucurbit[7]uril (CB7) and *p*-sulfonatocalix[4]arene (CX4) and dapoxyl and aminomethyl-substituted 2,3-diazabicyclo[2.2.2]oct-2-ene (DBO) as fluorescent dyes (see Fig. 10 for structures of hosts and Fig. 11 for structures of dyes). Both, CB7 and CX4 bind the zwitterionic amino acid substrates very weakly and the cationic decarboxylation products more strongly, but dapoxyl complexation by CB7 resulted in a fluorescence increase, while CX4 forms a non-emissive inclusion complex with DBO. This resulted in an overall OFF-response for the CB7/dapoxyl-based assay (Fig. 13a) and an ON-response for the DBO/CX4-based assay (Fig. 13b). It is noteworthy, that the host does not need to be exceptionally selective to successfully set up a tandem assay, but that a relative selectivity to discriminate substrate and product often suffices. The assay could be therefore universally applied to lysine, histidine, ornithine, tyrosine, and tryptophan decarboxylases, and the possibility to obtain time-resolved fluorescent kinetic traces were employed to extract enzyme kinetic parameters such as the Michaelis–Menten constants (*K*_m_). Subsequently, a refined variant of an ornithine decarboxylase assay was reported by us using cucurbit[6]uril (CB6) and *trans*-4-[4-(dimethylamino)styryl]-1-methylpyridinium iodide (DSMI) as a reporter pair, which was shown to be compatible with typical settings in HTS assays in the pharmaceutical industry (see Section 6).^60^

**Fig. 14:**
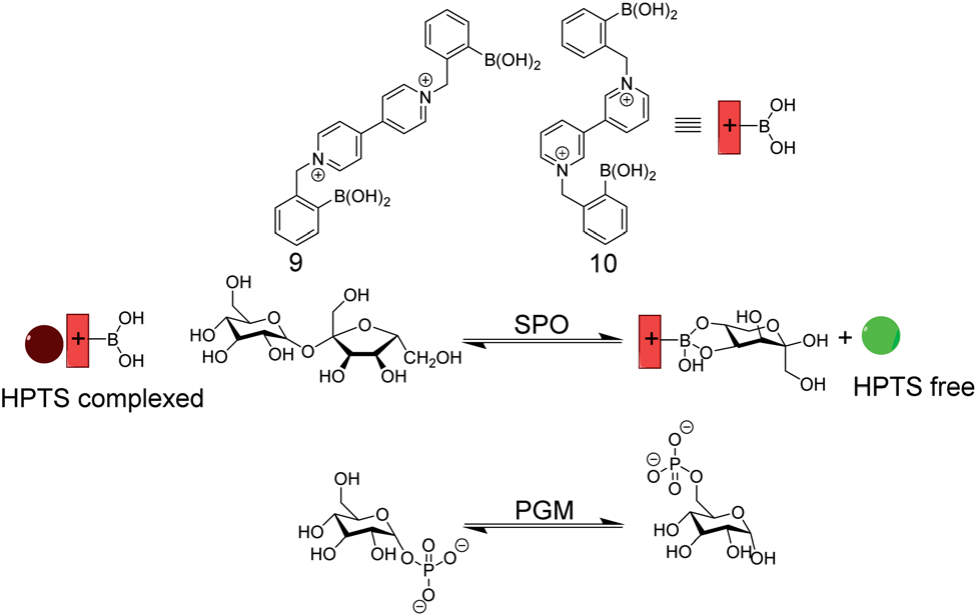
Enzyme assay with self-assembled carbohydrate chemosensors 9 and 10 with HPTS as a fluorescent dye (see Fig. 11 for dye structure). Sucrose phosphorylase (SPO) activity was monitored with 9, which selectively binds fructose that is generated by glucosyl transfer from sucrose to orthophosphate. The chemosensor 10/HPTS binds glucose-6-phosphate stronger than glucose-1-phosphate, which enabled monitoring of phosphoglucomutase (PGM) activity by HTPS displacement from 10 by the product glucose-6-phosphate.

Singaram and co-workers reported IDAs for selective sensing of carbohydrates and respective enzyme assays (Fig. 14).^61–64^ The receptors 9 and 10 are based on phenylboronic acids attached to a viologen unit and 8-hydroxypyrene-1,3,6-trisulfonic acid trisodium salt (HPTS) served as the indicator dye. The positively charged receptors bind with negatively charged HPTS through electrostatic interactions, which led to quenching of the HPTS fluorescence. Sucrose phosphorylase (SPO) catalyses the transfer of the glucose residue of sucrose to orthophosphate resulting in glucose-1-phosphate and fructose. The receptor 9 binds fructose selectively such that the enzyme reaction product fructose displaces HPTS from 9 leading to a recovery of the HPTS fluorescence in response to SPO activity. The constitutional isomer 10 can discriminate glucose-1-phosphate and glucose-6-phosphate, whereby the latter showed a stronger binding to 10. This enabled monitoring the isomerization reaction catalysed by phosphoglucomutase with the self-assembled 10/HPTS chemosensor.

The most recent example of a product-selective tandem enzyme assay involved the specific detection of steroids by CB7 and CB8.^65^ It was found that nandrolone had nanomolar affinity to CB8, whereas the affinity of nandrolone 17-propionate was lower by a factor of *ca.* 5. Therefore, hydrolysis of the low affinity propionate ester by pig liver esterase (PLE) was monitored with a CB8-based chemosensor.

### 4.3 Substrate-selective supramolecular tandem enzyme assays

**Fig. 15:**
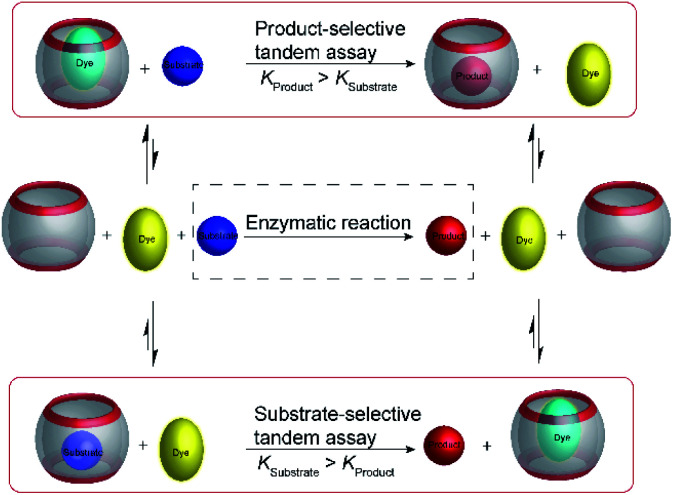
Schematic representations of product-selective and substrate-selective tandem enzyme assays. In a product-selective tandem enzyme assay (top), the product binds more strongly and displaces the fluorescent dye during the course of the enzymatic reaction. In a substrate-selective tandem enzyme assay (bottom), the substrate binds more strongly and the dye can bind to the macrocycle when the substrate is converted into a weaker binding product. Thereby, it is important to consider that complexation of the substrate may affect the enzymatic reaction. This potentially detrimental influence can be reduced by selecting assay conditions, in which sufficient free substrate exists in solution and only a minor fraction actually displaces the dye from the receptor at the beginning of the reaction.

Product-selective tandem enzyme assays are very intuitive and easy to design, when the net charge is increased during the enzymatic reaction, because water-soluble macrocyclic receptors rely on weak interaction involving charges for selective binding (anion or cation receptors).^26^ A change in the net charge of a molecule during an enzymatic reaction is very common, but many enzymatic reactions alter the net charge in the “wrong” direction for a product-selective assay. When the net charge of a molecule is decreased during the reaction, a host would be required that binds more strongly with a less charged product, which is actually very challenging to design. It thus appeared very desirable to establish “substrate-selective tandem assays”, in which the substrate has a higher binding affinity to the macrocycle than the product (Fig. 15).

One of the important aspects of a substrate-selective tandem enzyme assay is that the substrate is complexed by the receptor and may, thus, not be recognized by the enzyme. In extreme cases, the enzymatic reaction may be even fully inhibited.^17,66^ As a remedy, the conditions in a substrate-selective tandem enzyme assay may be chosen in such a way that only a small fraction of the substrate is actually bound (*e.g.* 10–20% of the total substrate concentration), whereas the major fraction of the substrate is uncomplexed and free in solution, where it can be accessed by the enzyme. The ideal condition for substrate-selective assay is met, when the substrate binds more strongly to the receptor than the product, but not too strongly to be fully complexed. During the course of the enzymatic reaction, the dynamic substrate binding will constantly replenish free substrate in solution, which has been removed from the equilibrium by conversion into the product.^67^

It is noteworthy that supramolecular tandem assays can be continuously monitored in the substrate-selective mode, which is commonly not possible with antibody-based enzyme assays. The reason is that antibodies have commonly much slower dissociation rates than supramolecular receptors.^68^ To allow full equilibration of a competitive binding equilibrium with antibodies requires incubation times of several minutes to hours, which is much slower than the enzymatic reaction and prevents a continuous monitoring. In contrast, exchange of supramolecular host–guest complexes proceeds within microseconds to milliseconds, such that the enzymatic reaction is rate-limiting and not the establishment of the binding equilibrium.

**Fig. 16:**
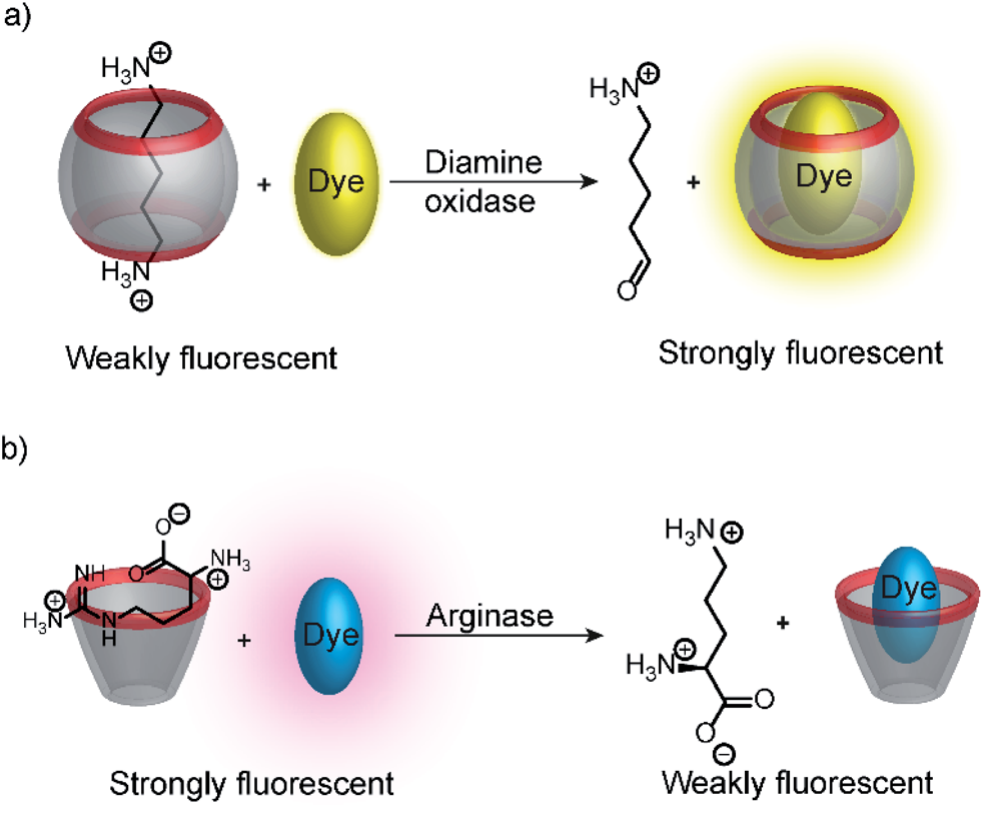
Substrate-selective supramolecular tandem enzyme assays. (a) Diamine oxidase converts the doubly positively charged substrate cadaverine, which is strongly bound by the cation receptor CB7, into singly positively charged 5-aminopentanal, which has a weaker affinity. (b) Arginase hydrolyses the guanidinium group of arginine to afford the non-proteinogenic amino acid ornithine. The latter has a weaker affinity to CX4 as a supramolecular receptor.

Substrate-selective tandem enzyme assays were initially realized with two different enzymatic reactions: the hydrolysis of the guanidinium group of arginine into ornithine by arginase and the conversion of cadaverine into 5-aminopentanal by diamine oxidase (Fig. 16).^67^ The CX4/DBO reporter pair was used for arginase and CB7/acridine orange (AO) was used for diamine oxidase. In both cases, the substrates had a higher affinity for the hosts than the products (6400 M^−1^ for arginine *vs.* 500 M^−1^ for ornithine and 4.5 × 10^6^ M^−1^ with cadaverine *vs.* 1.1 × 10^5^ M^−1^ with 5-aminopentanal), and the time-resolved fluorescence traces showed the expected switch-OFF and switch-ON response after enzyme-induced host–dye complex formation, respectively.

However, to extract meaningful enzyme kinetic parameters from fluorescence progress curves in substrate-selective assays, several precautions need to be considered. For example, complex formation of substrates with host molecules were often used to prevent undesirable enzymatic degradation, such that potential inhibitory effects of the host need to be considered.^66,69^ Moreover, progress curves of substrate-selective tandem assay may show a lag phase, in which the substrate is converted into the product without any observable change in fluorescence. This happens, when the concentrations of the reporter pair are too low for the selected substrate concentration, such that a large fraction of substrate remains in the reaction mixture although the enzymatic reaction has already significantly progressed. These effects are, however, not detrimental for the determination of inhibition constants; once appropriate concentrations of substrate and reporter pair that give an immediate response have been identified, the inhibitory effect is reflected in decreasing rates of initial fluorescence changes, which can be used to reliably determine inhibition constants. A comprehensive guide for the optimization of tandem enzyme assays including a thermodynamic and kinetic analysis has been published previously.^26^

**Fig. 17:**
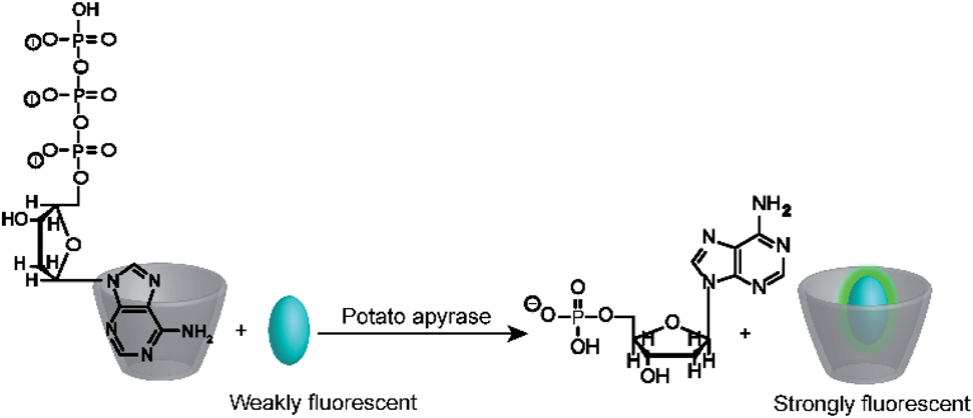
Substrate-selective tandem enzyme assay for monitoring ATP-dependent enzymes. The positively charged octakis(6-deoxy-6-amino)-γ-cyclodextrin binds the more negatively charged ATP more strongly than AMP, which enables monitoring of the hydrolysis of ATP by potato apyrase.

It is interesting to note, that substrate-selective assays were subsequently more widely adapted by the supramolecular analytical chemistry community than the much simpler product-selective assays. For example, Nau and co-workers tested several reporter pairs with the goal to monitor the hydrolysis of ATP.^70^ The reporter pairs included, among others, 2-anilinonaphtalene-6-sulfonate (ANS) in combination with octakis(6-deoxy-6-amino)-γ-cyclodextrin and 8-hydroxy-1,3,6-pyrene trisulfonate (HPTS) with a cyclophane (see Fig. 10 and 11 for structures). These anion receptors showed a higher affinity for the substrate ATP than for AMP, which enabled monitoring of potato apyrase activity in a switch-ON (amino-γ-cyclodextrin/ANS) and switch-OFF (cyclophane/HPTS) assay (Fig. 17). Moreover, the two reporter pairs were complementary with respect to the substrate affinity. With the high, micromolar affinity of amino-γ-cyclodextrin, substrate concentrations below the Michaelis–Menten constant, *K*_M_, of potato apyrase could be used, which is ideal for screening activators and inhibitors, whereas the low, millimolar affinity of cyclophane allowed to use substrate concentrations much above the *K*_M_, which is ideal to assess the maximal initial rate, *v*_max_, and thus enzyme purity (*e.g.* in quality control in enzyme production). Enzyme activation could be successfully confirmed with divalent metal ions (Ni^2+^, Mg^2+^, Ca^2+^, and Mn^2+^) and the extracted *v*_max_ values were in agreement with literature-known initial reaction rates. Finally, the transferability to other nucleotide triphosphates (GTP, TTP and CTP) was also demonstrated.

**Fig. 18:**
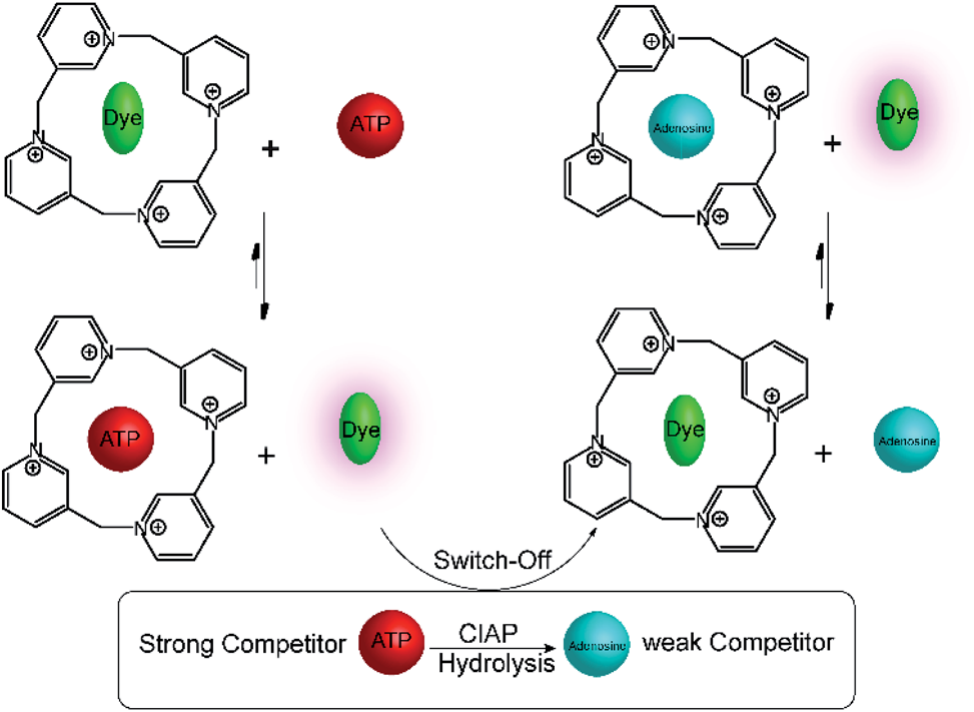
Substrate-selective tandem enzyme assays for calf intestinal alkaline phosphatase (CIAP) using calixpyridinium/PTSA as reporter pair. CIAP hydrolyses ATP, which binds strongly to the positively charged calixpyridinium receptor, into uncharged adenosine, which shows no interaction. The fluorescent dye PTSA can subsequently bind to the calixpyridinium receptor affording a fluorescence decrease during the course of the enzymatic reaction.

A related calixpyridinium-based tandem assay for alkaline phosphatase (ALP) was reported by Kui Wang and co-workers.^71^ Calixpyridinium, a positively charged water soluble macrocyclic host, which is bind with 1,3,6,8-pyrenetetrasulfonic acid tetrasodium salt (PTSA) led to a fluorescence quenching. Also, ATP and AMP have different binding affinity with calixpyridinium. Enzymatic transform of ATP into AMP can be easily monitored by calixpyridinium/PTSA reporter pair (Fig. 18), because substrate and product have different binding affinity to macrocycle. Addition of calf intestinal alkaline phosphatase (CIAP) into the reaction mixture containing calixpyridinium/PTSA and ATP, in which CIAP catalysis hydrolysis of ATP, resulted in decrease in fluorescence due to displacement of product (AMP) by the high affinity dye. Stepwise dephosphorylation with ADP as an intermediate unable to follow by the host calixpyridinium because the substrate ATP and intermediate product ADP have almost same binding affinity to the calixpyridinium (*K*_a_ = *ca.* 1 × 10^4^ M^−1^). The dependence of the initial reaction rate on varying substrate concentration experiments were carried out below the *K*_m_ value of the enzyme, and showed typical linear dependence of initial reaction rates on the substrate concentration. Also, Lineweaver–Burk plot gives *K*_m_ of (105 ± 21) μM.

ALP is known to have a broad substrate tolerance and thus cleaves many different phosphate ester bonds. Guo and co-workers could thereby monitor the dephosphorylation of pyridoxal-5′-phosphate (PLP) into pyridoxal and phosphate by ALP.^72^ The calix[5]arenes of the Guo group show very interesting molecular recognition properties.^39,73,74^ and a guanidinocalix[5]arene (G-CX5, see Fig. 10) bound fluorescein (FL) with micromolar affinity (*K*_a_ = 7.0 ± 2.1) × 10^6^ M^−1^ and strong fluorescence quenching. This afforded G-CX5/FL as a new reporter pair for anions, which was able to bind PLP with micromolar affinity (*K*_a_ = 2.0 ± 0.5) × 10^6^ M^−1^, whereas phosphate has millimolar affinity (*K*_a_ = 7.9 ± 0.9) × 10^4^ M^−1^ with G-CX5. Addition of ALP to a mixture of PLP and the reporter pair gave the expected switch-off fluorescence response and the initial reaction rates were found to be linearly dependent on the initial PLP concentration. This was used for the quantification of PLP with an excellent limit of detection of (26.5 ± 0.6) nM.

**Fig. 19:**
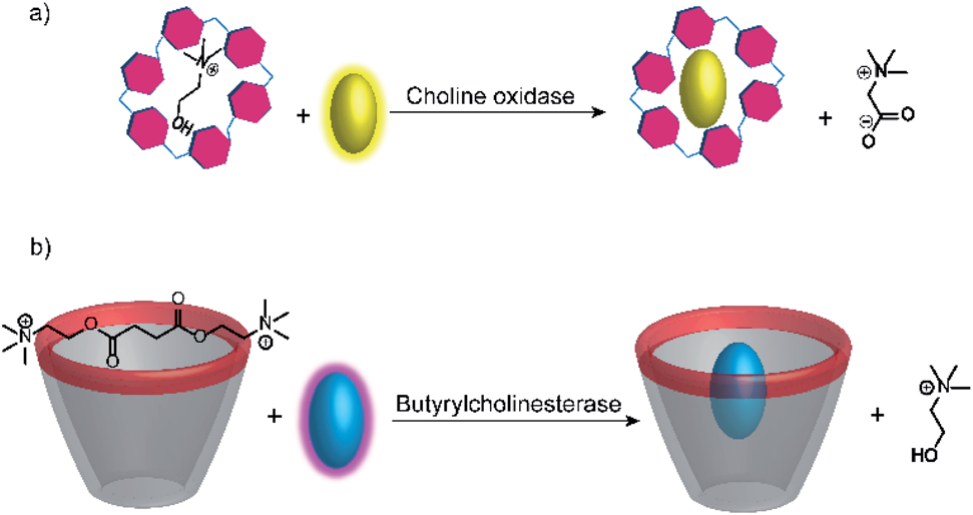
Substrate-selective supramolecular tandem enzyme assays measuring (a) choline oxidase activity with a per-carboxylated pillar[6]arene and AO as reporter pair (b) butyrylcholinesterase activity based on CX4/LCG as reporter pair. In both cases, the host is occupied by the substrate and the dye is fluorescent free in solution. After conversion of the substrate, the low-affinity product cannot displace the dye leading to complex formation and quenching.

Further substrate-selective tandem enzyme assays were developed for choline oxidase (CO) by Jie Yang and co-workers^75^ and by Guo and Liu for butyrylcholinesterase (BChE)^76^ by using cation receptors (Fig. 19). The group of Jie Yang showed for the first time the usefulness of pillararenes in tandem enzyme assays.^75^ The pillar[6]arene WP6 (see Fig. 10) formed a suitable reporter pair with AO resulting in fluorescent quenching and a colour change from yellow to red indicative of charge-transfer interactions. Choline showed a higher binding affinity (*K*_a_ = (5.26 ± 0.36) × 10^4^ M^−1^) than the choline oxidase product betaine, which afforded a product-selective switch-OFF tandem assay. Guo and Liu selected several sulfonated calixarenes based on the known recognition motif of the trimethylalkylammonium group of cholines for the possibility to discriminate succinylcholine and choline.^76^ They identified CX4 as the most suitable host and combined it with lucigenin (LCG, Fig. 11) to form the strongly quenched CX4/LCG reporter pair. CX4 was found to bind succinylcholine with low micromolar affinity and with 10 fold higher affinity than choline. Since BChE also hydrolyses succinylcholine into choline, BChE activity could be followed allowing even the determination of the *K*_M_ value of BChE despite the limited range of accessible substrate concentrations.

Very recently, a supramolecular tandem enzyme assay for flavin monooxygenase 3 (FMO3) has been reported.^77^ The enzyme FMO3 oxidizes the amine of trimethylamine (TMA) to afford trimethylamine *N*-oxide (TMAO), which is a significant risk factor in cardiovascular thrombotic events. It was found that the substrate TMA has a higher binding affinity to CX4 than the product TMAO, which suggested the use of a CX4-based reporter pair. Several dyes, namely LCG, methylene blue, pyronine, and oxazine 1 (OX1) were screened for their compatibility with the assay conditions, among which OX1 (see Fig. 11 for dye structure) was found to be most tolerant towards the presence of NADPH and NADP^+^ as well as TMAO. In the CX4/OX1 reporter pair, the fluorescence of OX1 was quenched by a factor of *ca.* 5 and the binding constant was sufficiently high (1.4 × 10^4^ M^−1^). Consequently, the fluorescence is decreased in the course of enzymatic reaction, because the consumption of TMA enabled the re-encapsulation of OX1 by CX4. This has been used to identify a series of FMO3 inhibitors from a traditional Chinese medicine composed of eleven herbs with known antithrombotic activity (see Section 6.1).

### 4.4 Enzyme-coupled assays and domino assays

**Fig. 20:**
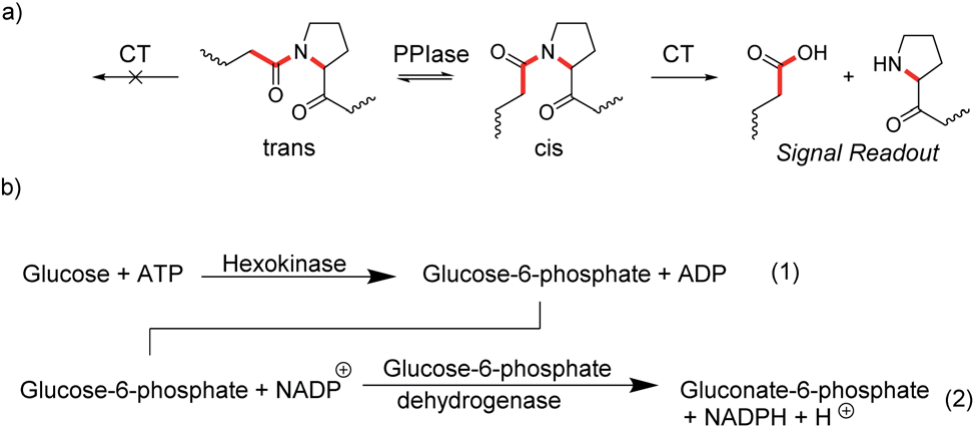
Coupled-enzyme assays for enzymatic reactions that are otherwise difficult to monitor. (a) *cis*–*trans* isomerization of the secondary amide bond at proline residues is catalysed by peptidylprolyl isomerase (PPIase). Subsequent selective cleavage of the *cis* form by excess chymotrypsin (CT) is easy to monitor spectroscopically with fluorogenic or chromogenic substrates. (b) Hexokinase activity (1) is monitored by oxidizing the product glucose-6-phosphate with an excess of the NADP^+^-dependent glucose-6-phosphate dehydrogenases (2). The conversion of NADP^+^ into NADPH is followed by an increase in the absorbance at 340 nm.

In many enzyme assays and biosensors, more than one enzyme is used. This strategy is classically applied when it is difficult to obtain an output signal from one of the enzymatic reactions. For example, peptidylprolyl isomerases (PPIases) are enzymes, which catalyze the *cis*–*trans* isomerization surrounding the proline amide bond (Fig. 20a) and which play important roles in protein folding.^78^ Such isomerization reactions are notoriously difficult to monitor by absorption or fluorescence spectroscopy. However, the protease chymotrypsin has a much higher catalytic efficiency for the *cis* form relative to the *trans* form of the proline bond and hydrolysis of peptide bonds are particularly easy to monitor by spectroscopy.^79^ The consequence is to set up an enzyme-coupled enzyme assay, in which the isomerization reaction is rate-limiting, such that every peptide with a *trans* proline amide bond is immediately cleaved resulting in a spectroscopic signal change. The remaining *cis* prolines are then converted into *trans* proline by PPIase as a rate-limiting step and the signal change from hydrolysis directly reflects the activity of PPIase.

Another popular strategy is coupling an enzymatic reaction that is difficult to monitor with NAD(P)H-dependent dehydrogenases. The reduction of NAD(P)^+^ into NAD(P)H and *vice versa* can be easily monitored by the absorption of NAD(P)H at 340 nm (*ε* = 6300 M^−1^ cm^−1^).^80^ This enables, for example, to monitor the reaction of hexokinase by converting the hexokinase product glucose-6-phosphate into gluconate-6-phosphate by the NADP^+^-dependent glucose-6-phosphate dehydrogenase and the increase in absorption due to formation of NADH (Fig. 20b).^81^ Kinetic analysis of the enzyme progress curves may become quite involved, when the enzyme-coupled enzyme assays cannot be ideally set up due to limitations in the enzyme kinetic parameters of the involved enzymes, or when the enzyme used for detection is not available in sufficiently large quantities to be used in excess amounts. However, generation of a measurable optical output signal is commonly unproblematic regardless of the actual enzyme kinetic parameters and concentrations.^81^

**Fig. 21:**
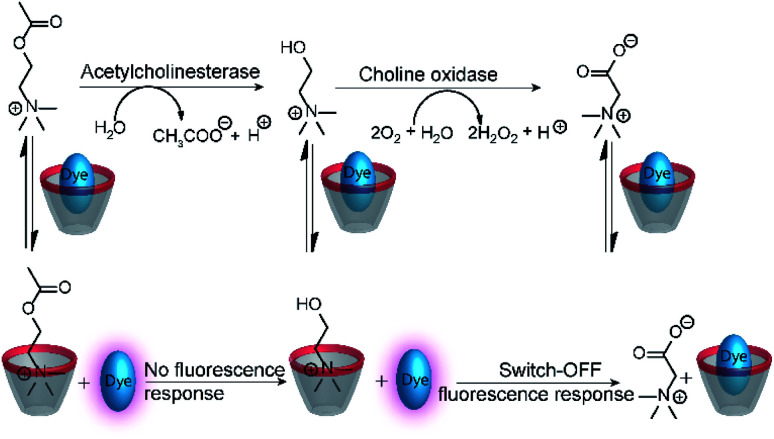
Monitoring of acetylcholinesterase activity by an enzyme-coupled tandem enzyme assay. Conversion of acetylcholine into choline gives no response of the CX4/LCG reporter pair, because the affinity of acetylcholine and choline is very similar. Subsequent conversion of choline into betaine by choline oxidase gives the desired fluorescence response that allows to follow acetylcholine esterase activity in presence of excess choline oxidase.

The versatility of enzyme-coupled enzyme assays is also very attractive to expand the scope of supramolecular tandem enzyme assays. The use of multistep enzymatic reactions in tandem assays was shown with an elegant model system based on the CX4/LCG reporter pair (Fig. 21).^82^ CX4 is incapable of discriminating between acetylcholine and choline, which prevents its use in a tandem assay for acetylcholinesterase (AChE). Therefore, an enzyme-coupled tandem assay was conceived using the transformation of choline to betaine by choline oxidase. The cation-responsive reporter CX4/LCG was able to distinguish between choline and betaine due to the different charge status of these two molecules. While choline is cationic, betaine is zwitterionic and has a much lower binding affinity to the cation receptor CX4. With an excess amount of choline oxidase, the enzymatic reaction of AChE became rate-limiting. This allowed the determination of enzyme kinetic parameters of AChE, which showed literature-known trends.

**Fig. 22:**
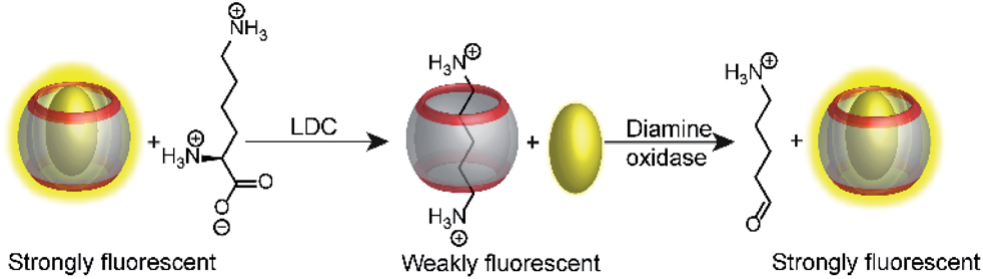
Consecutive monitoring of two enzymatic reactions (tandem domino assay) win ON–OFF–ON signal modulation. Decarboxylation of lysine to the stronger binder cadaverine leads to dye displacement and fluorescence switch-OFF. Subsequent addition of diamine oxidase turn the fluorescence ON, because the oxidation product cannot efficiently compete with dye binding.

An interesting way to combine two enzymatic reactions and a reporter pair has been coined “domino-tandem assay”, in which a two sequential enzymatic reactions and a single reporter pair afforded an ON–OFF–ON signal pattern (Fig. 22).^67^ Essentially, the demonstrated domino assay is a sequence of a product-selective and a substrate-selective assay and was realized with the CB7/AO reporter pair and the enzymes lysine decarboxylase and diamine oxidase. In detail, lysine is a weak competitor and the CB7/AO complex renders the mixture brightly fluorescent. Addition of lysine decarboxylase gives cadaverine and the fluorescence intensity decreases significantly, because the strong competitor cadaverine occupies the CB7 cavity. The sequential conversion of cadaverine into the weak competitor 5-aminopentanal by addition of diamine oxidase allows re-formation of the CB7/AO complex and the bright fluorescence is recovered.

### 4.5 Tandem enzyme assays using associative binding

**Fig. 23:**
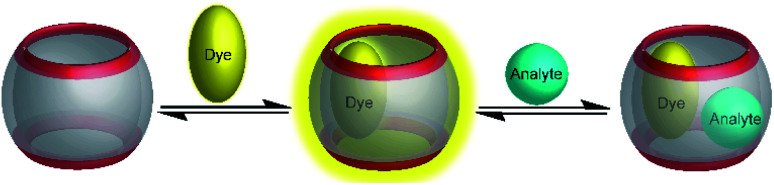
Associative binding assay for the detection of analytes. A sufficiently large macrocyclic host molecule binds first a dye; then, formation of a ternary complex with the analyte as a second guest modulates the optical properties, which allows sensing the analyte.

An alternative to indicator displacement is the construction of self-assembled chemosensors using ternary complexes composed of a macrocyclic host molecule, an optically active first guest (*e.g.* a fluorescent dye), and an analyte as a second guest (Fig. 23).^83^ For example, the cavity of CB8 is sufficiently large to accommodate two aromatic guest molecules to form a stable, ternary complex stabilized by aromatic π-electron donor–acceptor or charge transfer interactions within the hydrophobic cavity of CB8.^84^ The spectroscopic signal change immediately suggests its application as a chemosensor, for example with electron-poor methyl viologen for electron-rich naphthols and indoles as analytes, but the signal change was too weak for chemosensing.^85,86^

To enhance the sensitivity of associative binding assays electron-poor fluorescent dyes were found to be useful for the detection of electron-rich guests. In particular, inclusion of 2,7-dimethyldiazapyrenium (MDAP) by CB8 retained the bright fluorescence of MDAP, whereas binding of the analyte to the preformed CB8/MDAP complex resulted in strong fluorescence quenching of MDAP. This was successfully applied in homogeneous solution to the detection of a large variety of electron-rich aromatic guests, *e.g.* alkoxynaphthalenes, indole derivatives including the neurotransmitter tryptamine, and N-terminal phenylalanine residues in peptides.^40,83,87,88^

**Fig. 24:**
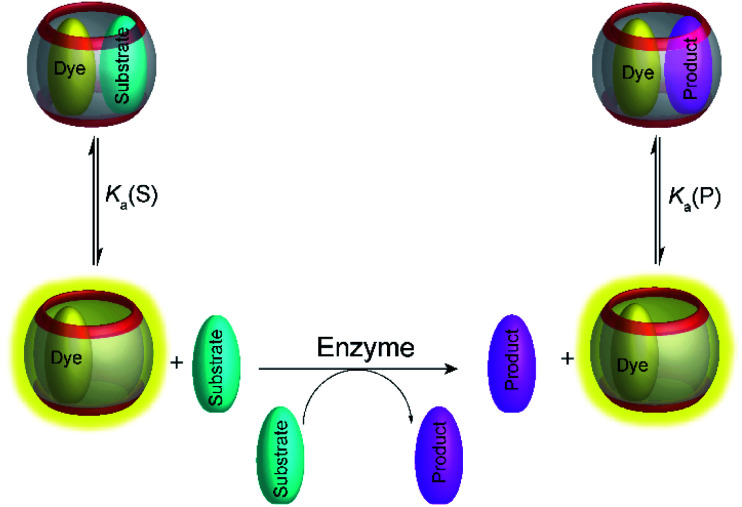
Schematic representation of supramolecular enzyme assays based on associative binding of ternary CB8 complexes. Due to the rapid exchange of CB8 ternary complexes, the substrate and product binding equilibria are established immediately on the time scale of the enzymatic reaction.

**Fig. 25:**
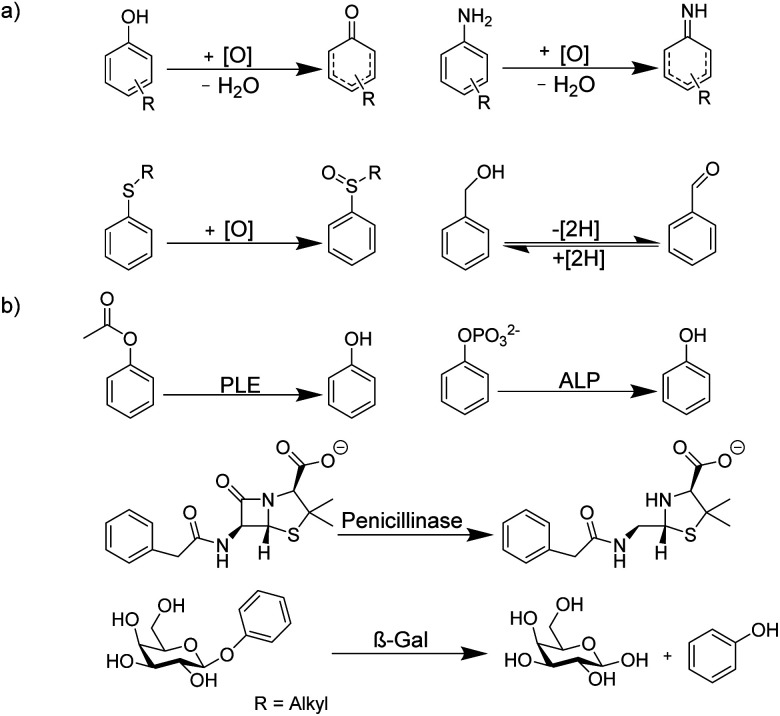
Supramolecular tandem enzyme assays based on associative binding of small aromatic guests to CB8 complexes with MDAP, MDPP, and/or PDI. Enzyme assays were demonstrated for (a) oxidoreductase activity and (b) hydrolase activity (see text for details about the enzymes).

Because of the rapid complex formation and dissociation kinetics of CB8 ternary complexes, associative binding is also attractive for real-time monitoring of enzymatic reactions (Fig. 24 and 25).^83^ Oxidoreductase activity (Fig. 25a) included the oxidation of various phenols, catechol, β-naphthol, aniline, and tryptophan by horseradish peroxidase (HRP), oxidation of tryptophan by lactoperoxidase (LPO), oxidation of 4-chlorophenol by laccase, and oxidation of thioanisole by chloroperoxidase (CPO). These reactions were successfully monitored with the CB8/MDAP chemosensor (except tryptophan and LPO, which was monitored with CB8/MDPP). Since the CB8/MDAP sensor was not affected by the presence of the co-substrates NAD^+^ or NADH, oxidation of *p*-methoxybenzyl alcohol and reduction of benzaldehyde by NADH/NAD^+^-dependent alcohol dehydrogenase (ADH) could be followed as well. In selected cases, the reactions were also monitored with CB8 complexes of perylenediimides (PDI) and dimethyldiazaperopyrenium (MDPP; see Fig. 11 for structures) as dyes, which afforded similar kinetic profiles and suggested that the chemosensors do not detrimentally affect the enzyme activities. The usefulness of associative tandem enzyme assays for enzyme activity screenings was also demonstrated with HRP and varying 4-substituted phenols as substrates, where more electron-rich phenols were oxidized more rapidly (Fig. 25a: R = –OMe > –Me > –H > –F), and by the pH dependence of tryptophan oxidation by HRP, which was consistent with the formation of different chemical intermediates at different pH.^89^ Moreover, the accelerated oxidation of chlorophenol in the presence of 2,2′-azino-bis(3-ethylbenzothiazoline-6-sulphonic acid) (ABTS) or *N*-hydroxy-phthalimide as redox mediators was successfully reproduced.

Apart from redox reactions, numerous enzymatic hydrolysis reactions were also monitored by associative binding. This included detection of pig liver esterase (PLE) with phenyl acetate, alkaline phosphatase (ALP) with phenyl and naphthyl phosphate, hydrolysis of the β-lactam ring of penicillin G by penicillinase, and hydrolysis of phenyl-β-d-galactopyranoside by β-galactosidase (β-Gal) with CB8/MDAP (Fig. 25b). The exopeptidase activities of carboxypeptidase A and leucine aminopeptidase (LAP) and the endopeptidase activity of pepsin could be monitored by CB8/MDPP, which is described in more detail in Section 5.1.

Overall, the wealth of different enzymatic reactions investigated with associative reporter pairs demonstrated the versatility of this new enzyme assay platform. It is interesting to note, that the substrate and product affinities as well as the quenching efficiencies have not been investigated in detail for all reported enzyme activities. Although it transpired from the fluorescence progress curves of the enzymatic reactions that more electron-rich second guests were more efficiently bound and/or quenched by the electron-poor CB8/dye chemosensors, it is interesting to conceive that tandem assays based on associative binding could be also potentially realized when substrate and product have the same affinity (*K*_a_ (S) = *K*_a_ (P)), but show different spectroscopic responses, for example a different quenching efficiency of the ternary host/dye/substrate and host/dye/product complexes.

## 5. Supramolecular enzyme assays with peptides and proteins

In the preceding sections, we have introduced the various concepts, which enable the monitoring of enzymatic reactions with label-free substrates by using supramolecular receptors. From the viewpoint of enzyme assay development, some of the investigated enzymes are easy to monitor with other types of assays. This includes, for example, unspecific hydrolases such as ALP, PLE, or β-Gal, for which numerous fluorogenic and chromogenic substrates are commercially available. However, the simplicity of supramolecular enzyme assays is unmet by other assays for other types of enzymes. For example, amino acid decarboxylases have a very narrow substrate specificity and easily distinguish homologues of amino acids (*e.g.* ornithine and lysine). Consequently, decarboxylases do not tolerate major substrate modifications such as the introduction of chromogenic or fluorogenic dyes.^90^ In principle, antibody-antigen recognition can also be used to detect label-free enzyme reaction products or substrates,^19^ but it is particularly challenging to raise antibodies against low-molecular weight compounds such as amino acids. As a consequence, label-free supramolecular enzyme assays present an attractive alternative to conventional enzyme assays.

### 5.1 Supramolecular protease assays

As mentioned above, many unspecific hydrolases are easy to monitor, because fluorogenic or chromogenic residues can be easily introduced without losing substrate specificity. However, many other peptidases and proteases have a much narrower substrate specificity and require extended amino acid sequences for successful substrate recognition.^11,91^ The most common approach to assess these peptidases and proteases involves double-labelling of the peptide recognition sequence at remote sites. This may become synthetically cumbersome and is not applicable to exoproteases such as carboxy- and aminopeptidases (see below), which cleave off a natural C-terminal or N-terminal amino acid.^11–13^ But even if double-labelling can be performed, some proteases such as clostridial neurotoxins are very sensitive towards substrate labelling and the substrate activity is largely sacrificed by synthetic modifications.^92,93^ Supramolecular enzyme assays can therefore also present an attractive, label-free alternative for peptide-based substrates; the prerequisite is that the supramolecular receptor can distinguish between the intact peptide and the peptide fragments after hydrolysis.

**Fig. 26:**
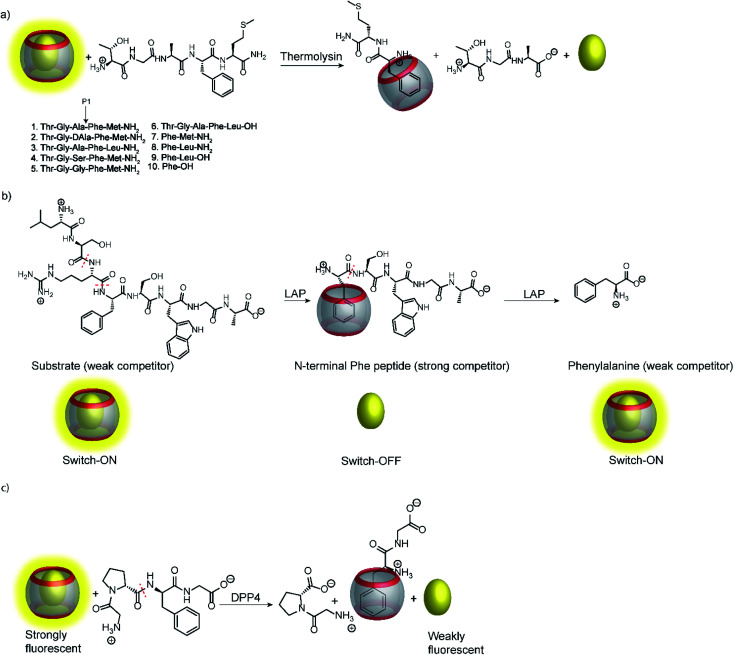
Supramolecular tandem enzyme assays involving recognition of an N-terminal Phe residue in peptides by CB7. (a) Continuous fluorescence enzyme assay for the protease thermolysin based on the recognition of a liberated N-terminal Phe residue by the CB7/AO reporter pair. (b) ON–OFF–ON signal modulation during successive cleavage of N-terminal amino acid residues by LAP converting a weak competitor, first, into strong competitor, and then, into a weak competitor. (c) Detection of dipeptidyl peptidase 4 (DPP4) activity after liberation of H–Phe–Gly–OH from H–Gly–Pro–Phe–Gly–OH.

Peptide recognition motifs have been explored for numerous macrocyclic host molecules including calixarenes and cucurbiturils.^94,95^ For example, the amino acid phenylalanine has only moderate affinity to CB7 as an internal peptide residue (*ca.* 10^4^ M^−1^), whereas an N-terminal Phe binds much more strongly due to additional ion–dipole interactions with the positively charged ammonium group (*K*_a_ > 10^6^ M^−1^).^96,97^ This differential binding affinity has been exploited in a label-free enzyme assay for thermolysin (Fig. 26a).^98^ Thermolysin is an endoprotease, which cleaves the amide bond at the α-nitrogen atom of Leu and Phe residues and liberates a peptide with an N-terminal, positively charged Leu or Phe residue. Overall, the binding affinity of six different enkephalin-type peptide substrates (Fig. 26a, 1–6) and their respective cleavage products (Fig. 26a, 7–9) to CB7 was investigated, which revealed a lower affinity of the substrates (*K*_a_*ca.* 10^4^ M^−1^) compared to the products (*K*_a_ > 10^7^ M^−1^) and allowed to monitor substrate cleavage in a product-selective, label-free enzyme assay with the reporter pair CB7/AO.

Interestingly, the binding affinity determinations revealed a different affinity of the diastereomeric substrates 1 and 2 with l- and d-Ala residues in the P1 position with CB7. Such a diastereodifferentiation of dipeptides was previously noted for Phe–Leu with l-Leu and d-Leu.^99^ With respect to enzyme specificity, substrate 1 with an l-Ala residue was rapidly hydrolysed, whereas cleavage of peptide 2 with d-Ala was insignificant, which is in accordance with the known specificity of thermolysin.^100^ Even more interesting, a previously unreported exopeptidase activity of thermolysin has been discovered in the course of developing the supramolecular tandem assay. According to the reported substrate specificity of thermolysin, cleavage of substrate 3 should only occur at the Ala–Phe peptide bond, but not at the Phe–Leu bond, because the latter would involve cleavage of a C-terminal amino acid and, thus, exopeptidase activity. However, dye displacement from CB7 was much lower than expected for the product Phe–Leu–NH_2_, which prompted a more thorough investigation of the cleavage products by mass spectrometry. This revealed formation of H–Thr–Gly–Ala–Phe–OH as a product, which clearly showed that thermolysin can cleave C-terminal Leu and, thus, act as an exopeptidase. Noteworthy, such exopeptidase activities of endopeptidases remain undetectable with conventional double-labelling approaches in protease assays.^11^

Another interesting observation was an ON–OFF–ON signal modulation, when the peptide H–Leu–Ser–Arg–Phe–Ser–Trp–Gly–Ala–OH was cleaved by LAP in the presence of the reporter pair CB7/AO (Fig. 26b).^101^ LAP is an aminopeptidase with relatively broad substrate specificity and thus successively cleaves off N-terminal amino acid residues. After initial cleavage of several amino acid residues, the peptide fragment H–Phe–Ser–Trp–Gly–Ala–OH is formed. The N-terminal phenylalanine residue strongly binds to CB7 and gives the switch-OFF fluorescence response by AO displacement. Subsequently, LAP continues the hydrolysis and also cleaves off the N-terminal phenylalanine residue to afford H–Ser–Trp–Gly–Ala–OH. The low binding affinity of this peptide allows re-formation of the CB7/AO complex, which is indicated by a fluorescence switch-ON response. Reminiscent of the tandem domino assays (see Section 4.4), this signal generation pattern presents a combination of product-selective and substrate-selective molecular recognition of the peptide fragments generated by the same enzyme. The proposed mechanism was confirmed by detecting the respective peptide fragments by mass spectrometry (ESI-MS). In contrast, digestion of the substrate by trypsin yielded the product H–Phe–Ser–Trp–Gly–Ala–OH and the concomitant switch-OFF response without a subsequent regeneration of the fluorescence intensity.

Recognition of N-terminal Phe residues was subsequently exploited by Yong Bi and co-workers to develop an enzyme assay for dipeptidyl peptidase 4 (DPP4) based on CB7/AO (Fig. 26c).^102^ DPP4 cleaves off dipeptides from the N-terminus of peptides or small proteins (below 80–100 residues) with a preference for proline or alanine in the S1 position of the cleaved peptide bond.^103^ The tetrapeptide H–Gly–Pro–Phe–Gly–OH is an established substrate, which yields H–Phe–Gly–OH after enzymatic cleavage. The latter has a stronger binding affinity to CB7 than the substrate or the other cleavage fragment H–Gly–Pro–OH due to liberated N-terminal Phe, which results in a turn-OFF fluorescence response due to AO displacement. In addition, another two tetrapeptides were investigated, namely H–Gly–Pro–Gly–Gly–OH and H–Gly–Pro–Gly–Phe–OH. The results revealed that neither the substrate nor the product showed considerable binding with CB7 due to the lack of an N-terminal aromatic amino acid, which is required for strong binding with CB7. The authors demonstrated the potential of the assay for HTS applications and miniaturized the system with microplates. This allowed the investigation of enzyme kinetics, which indicated a linear dependence on the enzyme concentration and gave *K*_m_ = 282.6 μM by a Lineweaver–Burk plot. Also, a limit of detection (LOD) of 18.6 ng mL^−1^ was found for DPP4 and the specificity of the assay was confirmed with α-glucosidase, bovine serum albumin (BSA), lipase, α-chymotrypsin, carboxypeptidase Y and trypsin, which indicated a high selectivity of the assay towards DPP4.

The first supramolecular protease assay using calixarenes as host molecules was reported by the group of Dong-Sheng Guo.^73^ They found that insulin has μM affinity to the macrocycle CX4, which was exploited in a protease assay for pepsin using the CX4/LCG reporter pair. The peptide fragments resulting from the enzymatic cleavage by pepsin had a lower affinity than the substrate insulin resulting in a substrate-selective assay with a fluorescence switch-OFF response. The dependence of the initial reaction rate on substrate concentration was also investigated and gave a linear Lineweaver–Burk plot with a *K*_m_ value of (1.4 ± 0.3) μM. This assay may be useful in clinical diagnostics (see Section 6.2).

**Fig. 27:**
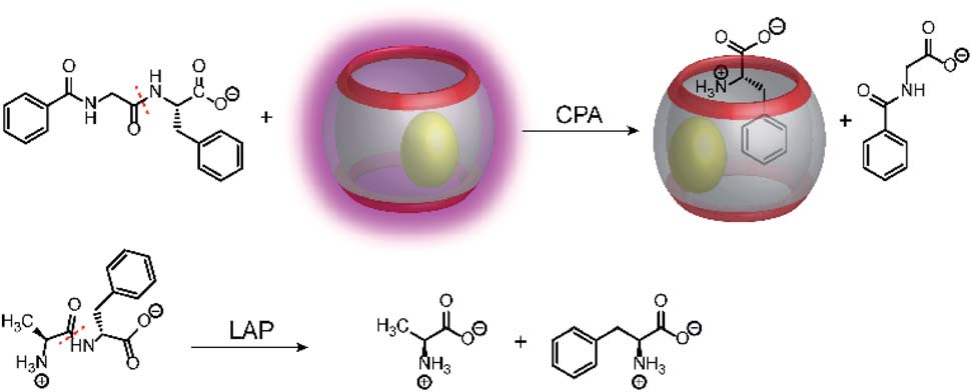
Monitoring amino acids released by C-terminal (CPA, panel a) or N-terminal (LAP, panel b) peptide hydrolysis with the CB8/MDPP reporter pair. CPA cleaves C-terminal amino acids releasing phenylalanine from hippuryl-l-phenylalanine. LAP cleaves N-terminal amino acids releasing phenylalanine from H–Ala–Phe–OH.

Supramolecular tandem protease assays were also shown based on associative binding with the CB8/MDPP reporter pair (Fig. 27).^83^ This included the exoproteases leucine aminopeptidase (LAP) and carboxypeptidase A (CPA) and the endoprotease pepsin. The CB8/MDPP reporter pair was used to monitor C-terminal hydrolysis of hippuryl-l-phenylalanine (=*N*-benzoyl-Gly-Phe-OH) by CPA and N-terminal hydrolysis of H–Ala–Phe–OH, H–Trp–Gly–Gly–OH, H–Gly–Trp–Gly–OH, and H–Trp–Leu_6_–H by LAP. As noted in Section 4.5, quenching efficiencies and binding affinities were not always clarified, but it can be assumed that the free aromatic amino acids phenylalanine and tryptophan had a higher binding affinity to the macrocycle CB8/MDPP complex than the amino acid side chains in the peptides. Ternary complex formation with CB8/MDPP reduced the fluorescence intensity of MDPP in response to the enzymatic reaction. As another demonstration, most proteins contains aromatic amino acids inside the protein hydrophobic core, which are thus unavailable for direct interaction with chemosensor due to steric shielding. Addition of relatively unspecific peptidases (*e.g.* pepsin) into a solution containing protein (*e.g.* bovine serum albumin (BSA)) lead to the degradation of the protein and loss of the its tertiary structure. The aromatic amino acids, which were previously in the hydrophobic core, can then interact with the chemosensor. This has been shown with BSA and pepsin at pH 2 in the presence of CB8/MDPP.

### 5.2 Supramolecular methyltransferase and demethylase assays

**Fig. 28:**
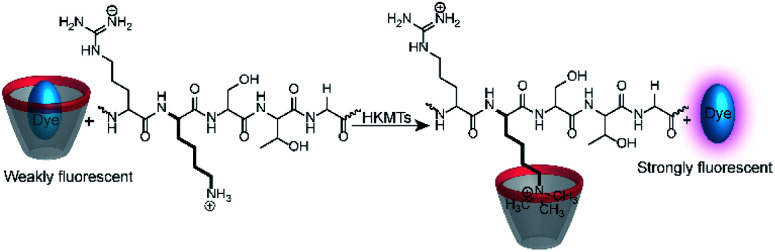
Monitoring the activity of lysine methyltransferases with the CX4/LCG reporter pair. The assay is based on the high affinity of trimethylalkylammonium residues to the CX4 receptor, which displaces the fluorescent dye LCG in the course of the enzyme-induced methylation of the lysine side chain.

Calixarenes are well-known for their high affinity towards trimethylalkylammonium groups (see the AChE assay in Section 4.4). This molecular recognition pattern also prompted the use of calixarene-based reporter pairs for an enzyme assay for histone lysine methyltransferases (HKMTs).^104^ HKMTs are responsible for methylation of lysine residues at particular sites of histone tails using (*S*)-adenosyl-l-methionine as a methyl donor (Fig. 28) and the HKMT class Dim-5 gives specifically trimethylated lysine residues at histone tails.

The binding constants of various methylated lysine derivatives increased from *K*_a_ < 10^3^ M^−1^ for Lys to *K*_a_ = 1.3 × 10^5^ M^−1^ for trimethylated lysine, such that an increase in the binding affinity was expected upon HKMT activity. The supramolecular tandem enzyme assay with the CX4/LCG reporter pair provided a switch-ON fluorescence response consistent with a higher affinity of the trimethylated product compared to the substrate containing an unmodified lysine residue. In an effort to determine enzyme kinetic parameters, the expected linear dependence of the initial reaction rates on varying enzyme concentrations at constant substrate concentrations was confirmed. However, the substrate concentration could only be varied within a narrow range and no enzyme kinetic parameters could be extracted. Within *ca.* 1 to 5 μM substrate concentration, an approximately linear dependence of the initial reaction rates suggested that the substrate concentrations were below the *K*_M_ of the enzyme.

**Fig. 29:**
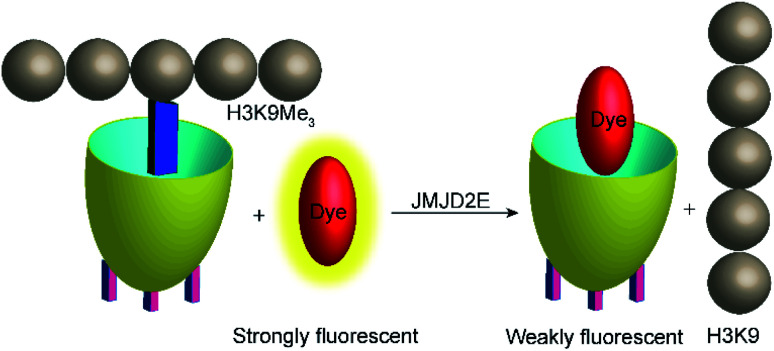
Monitoring the demethylation of the trimethylated lysine residue 9 of the substrate H3K9Me_3_ (see text for peptide sequence) by the histone demethylase JMJD2E. The cavitand 1 binds the trimethylammonium side chain of the Lys(Me)_3_ residue (blue rectangle), whereas the demethylated product H3K9 does not bind efficiently. The fluorescent dye FAMR will thus bind after demethylation leading to a fluorescence decrease as a response to the lysine demethylase activity of JMJD2E.

Demethylation reactions of lysine side chains in peptides were subsequently explored by Hooley and co-workers with the histone demethylase JMJD2E, which catalyses the demethylation of a histone H3 peptide fragment at lysine residue 9 of the substrate H3K9Me_3_ (peptide sequence: Ala–Arg–Thr–Lys–Gln–Thr–Ala–Arg–Lys(Me_3_)–Ser–Thr–Gly–Gly–Lys–Ala–Pro–Arg–Lys–Gln–Leu–Ala) (Fig. 29).^105^ They used cavitand 1, which is also known to bind trimethylalkylammonium ions, and the fluorescent dye FAMR as reporter pair (see Fig. 10 and 11 for structures). Binding of the reporter pair was based on the anchor group strategy,^106,107^ but actual quenching occurred due to aggregation of the FAMR/cavitand 1 complex. Binding titrations gave a dissociation constant of *K*_d_ = (17 ± 10) μM for FAMR/cavitand 1 and addition of H3K9Me_3_ to the reporter pair led to fluorescence enhancement due to the displacement of the dye from the cavity of cavitand 1 and concomitant deaggregation. When JMJD2E was added, a continuous decrease in fluorescence was observed, which originated from demethylation of the lysine residue in H3K9Me_3_ to afford the product H3K9.

In an extension of this work, site-selective post-translational modifications in H3 peptides at lysine residues in positions 4, 9, and 27 (H3K4Me_3_, H3K9Me_3_, H3K27Me_3_) could be distinguished.^108^ Therefore, a receptor array of cavitands 1 to 3 (Fig. 10) with FAMR (Fig. 11) as a dye was used in 96-well microtiter plates. The resulting fluorescence response was analysed by principal components analysis (PCA), which clearly indicated that the sensor array can discriminate the trimethylation in the different positions. The sensor array could also be used to confirm the site-selectivity of the JMJD2E demethylase and the lysine methyltransferase PRDM9.

### 5.3 Supramolecular kinase and phosphatase assays

**Fig. 30:**
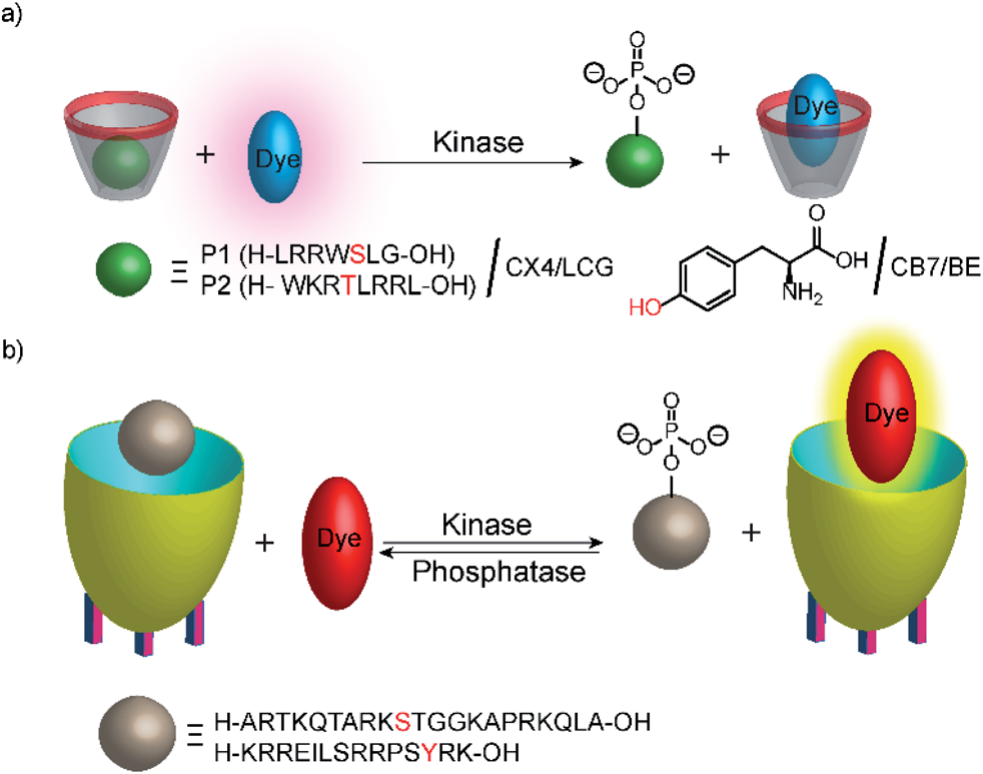
Monitoring phosphorylation and dephosphorylation with supramolecular tandem enzyme assays: (a) the CX4/LCG reporter pair was used to follow the activity of the serine kinase protein kinase A (PKA) with peptide P1 and the threonine kinase protein kinase C (PKC) with peptide P2. Phosphorylation introduces a negative charge and the phosphorylated peptide binds more weakly with the negatively charged CX4. (b) The cavitand 1 can be used with the dye DSMI to detect the unphosphorylated cationic peptide H3. Phosphorylation by a protein kinase A or aurora B kinase at the serine residue 10 reduces the binding affinity with the negatively charged cavitand. DSMI then displaces the peptides and gives a fluorescence increase.

Phosphatases and, in particular, kinases are important enzyme classes, which are often targeted in HTS applications in drug discovery.^14^ One possibility to monitor kinase activity is the use of a reporter pair that can discriminate the kinase co-substrate ATP from ADP that results from the transfer of the phosphate group (see Section 4.3). However, kinase and phosphatase assays can also be realized involving supramolecular reporter pairs, which discriminate phosphorylated and unphosphorylated peptides.^109^ This was shown with the two cationic peptides P1 (H-LRRWSLG-OH) and P2 (H-WKRTLRRL-OH), which bind with micromolar affinity to CX4 and efficiently displace LCG from the CX4/LCG reporter pair (Fig. 30a). The respective peptides, which were phosphorylated at the serine and threonine residue, had a much lower affinity, because the phosphate group reduces the overall positive charge of the peptides and, thus, decreases the binding affinity with the cation receptor CX4 significantly. Enzymatic phosphorylation of P1 and inhibition by *N*-ethylmaleimide was finally shown with the serine kinase protein kinase A (PKA). Noteworthy, dephosphorylation of the low-molecular weight substrate *O*-phospho-l-tyrosine (pTyr) by alkaline and acid phosphatase could also be monitored in the presence of CB7 and berberine (see Fig. 11) as a reporter pair at micromolar concentration of pTyr.^109^

In another study, the activity of kinases and phosphatases was explored with cavitand 1 (Fig. 10) and DSMI (Fig. 11).^110^ DSMI has a lower affinity to the cavitand (*K*_d_ = 23.1 μM) than the dye FAMR (*K*_d_ = 1.51 μM), which was used for following demethylation of the histone H3 peptide fragment H3K9Me_3_ (see Fig. 29 in Section 5.2). Consequently, the unmodified peptide H3 (*K*_a_ = 2.1 × 10^5^ M^−1^) could displace the lower affinity dye DSMI enabling detection of H3. Phosphorylated H3 at the serine residue 10 (H3S10p) had a further decreased binding affinity (*K*_a_ < 3000 M^−1^) enabling kinase detection in a substrate-selective tandem assay (Fig. 30).

The tandem assay was performed with protein kinase A (PKA) and aurora B kinase, which are both known to phosphorylate serine residues. The required buffer additives ATP, cAMP, and Mg^2+^ had no influence on the cavitand 1/DSMI reporter pair and the fluorescence increased as expected due to the phosphorylation of the serine residue in H3. The observed reaction rates were compared to reaction rates determined by MALDI-MS, which agreed very well. This assay was then used to explore the influence of posttranslational modifications of H3 on the PKA activity. This indicated that trimethylated H3K9Me_3_ and acetylated H3K9Ac were phosphorylated at comparable rates by PKA, whereas aurora B kinase phosphorylation was much slower with the dimethylated substrate H3K9Me_2_ compared to unmodified H3 in accordance with the literature. Noteworthy, the methylated lysine residues did not detrimentally interfere with detecting the phosphorylation reaction by the reporter pair as confirmed by MALDI-MS despite their stronger affinity to the cavitand 1. Finally, monitoring of dephosphorylation was shown with alkaline phosphatase and CREBtide, which was C-terminally modified with Lys and phosphorylated at the tyrosine residue, as substrate (peptide sequence: H–Lys–Arg–Arg–Glu–Ile–Leu–Ser–Arg–Arg–Pro–Ser–pTyr–Arg–Lys–OH).

### 5.4 Supramolecular tyrosinase assays

**Fig. 31:**
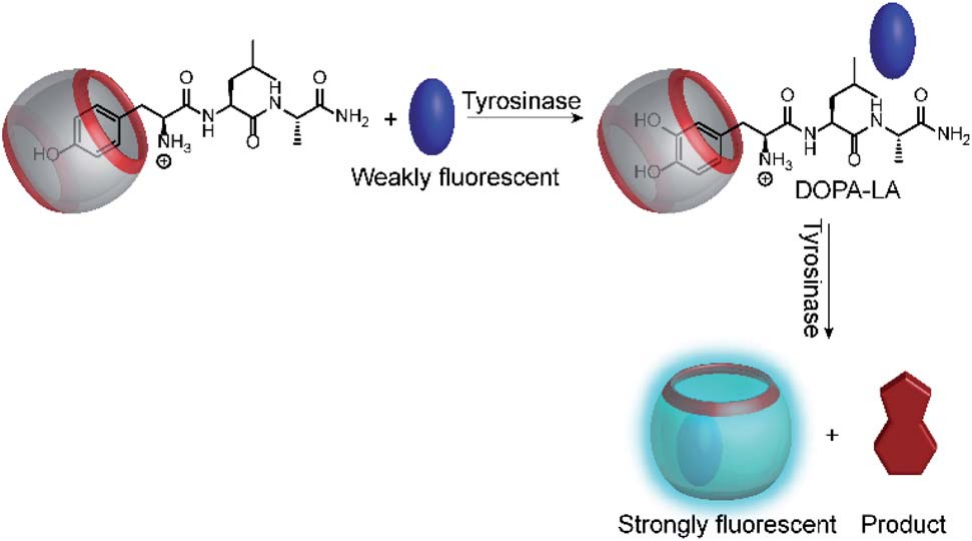
Tandem enzyme assay for tyrosinase with the CB8/ThT reporter pair.

Another very recent supramolecular tandem enzyme assay was reported for the enzyme tyrosinase by Cai and Li (Fig. 31).^111^ They explored a new reporter pair based on CB8 and thioflavin-T (ThT), which forms a 2 : 2 host–dye complex with a brightly fluorescent ThT excimer (*λ*_em_ = 570 nm).^112^ Tyrosinase accepts a broad variety of phenols and catechols as substrates,^113^ but Tyr and DOPA did not bind to CB8. Therefore, the authors selected the tripeptide H–Tyr–Leu–Ala–OH (YLA), which had been reported to bind to CB8 with high affinity (*K*_d_*ca.* 0.35 μM).^114^ Addition of tyrosinase into the substrate H–Tyr–Leu–Ala–H in the presence of CB8/ThT resulted in a fluorescence increase suggesting that the reaction product DOPA–Leu–Ala had a lower affinity enabling reformation of the CB8/ThT 2 : 2 complex. However, it was found that DOPA–Leu–Ala had a very similar binding affinity (*K*_d_*ca.* 0.31 μM) as the substrate. The authors thus concluded that the fluorescence increase resulted from the continued oxidation of DOPA–Leu–Ala to the respective quinones. The latter then form a complex product mixture with overall lower binding affinity than Tyr–Leu–Ala or DOPA–Leu–Ala.

## 6. Emerging applications of supramolecular enzyme assays

Within this final section, we are going to focus on applications of tandem assays. Since their infancy *ca.* 15 years ago, the applicability of supramolecular tandem enzyme assays has been widely demonstrated and nowadays includes inhibition studies and screening of compound libraries (Section 6.1) as well as the detection of enzyme activity for clinical diagnosis (Section 6.2) and microbial screenings (Section 6.3). The concept of supramolecular tandem enzyme assays can also be used to afford highly selective biosensors (Section 6.4) and can be transferred to the detection of enzyme activity by magnetic resonance imaging (MRI) as an alternative read-out to optical methods (Section 6.5).

### 6.1 Inhibitor screening

Already during the initial report on supramolecular tandem enzyme assays, inhibition constant determinations of known inhibitors have been included to demonstrate the utility of tandem enzyme assays for enzymological investigations or for drug screening applications (see Table 1 for a current overview). This included the arginase inhibitors *S*-(2-boronoethyl)-l-cysteine and 2-(*S*)-amino-6-boronohexanoic acid,^67,115^ inhibition of diamine oxidase by cyanide,^67^ inhibition of thermolysin by phosphoramidon,^98^ inhibition of acetylcholine and butyrylcholine esterase by tacrine and huperzine A,^76,82^ and the inhibition of the lysine methyltransferase HKMT Dim-5 by 1,10-phenanthroline.^104^ More recently, this utility of tandem enzyme assays was also demonstrated by Hooley and Zhong for inhibition of the lysine demethylase JMJD2E by the 2-oxoglutarate analogue 2,4-carboxypyridine^105^ and by Cai and Li for the tyrosinase inhibitor kojic acid.^111^

**Table 1:** Enzyme inhibitors that have been investigated with supramolecular tandem enzyme assays

#	Reporter pair	Enzymatic reaction	Inhibitor	IC_50_ or *K*_i_ (μM)	Ref.
1	CX4/DBO	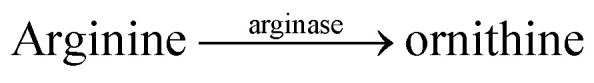	*S*-(2-Boronoethyl)-l-cysteine	3.7 ± 0.7	67
			2-(*S*)-Amino-6-boronohexanoic acid	0.2 ± 0.04	67
2	CB7/AO	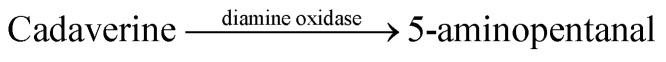	KCN	210 ± 110	67
3	CB7/AO	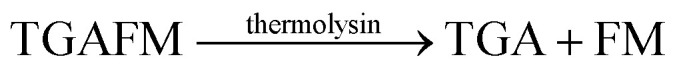	Phosphoramidon	(25 ± 0.4) × 10^−3^	98
4	CX4/LCG	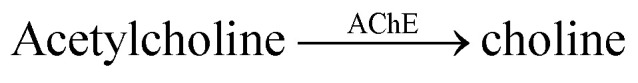	Tacrine	(20 ± 2) × 10^−3^	82
			Huperzine A	(120 ± 10) × 10^−3^	82
5	CX4/LCG	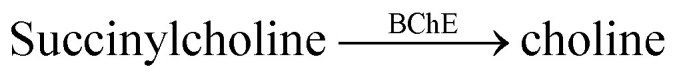	Tacrine	(10 ± 1) × 10^−3^	76
6	CX4/LCG	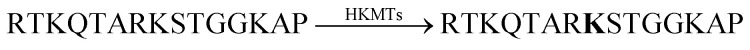	1,10-Phenanthroline	70 ± 20	104
7	Cavitand/FAMR	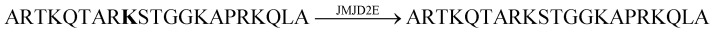	2,4-Dicarboxypyridine	7.4	105
8	CB8/ThT	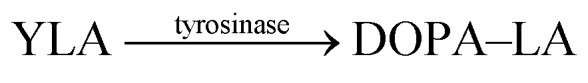	Kojic acid	30	111
9	CB7/dapoxyl	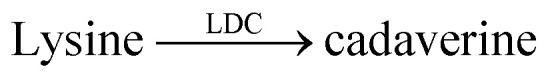	Perfluorinated carboxylic acids	2.8–291^a^	117
			Perfluorinated sulfonic acids	41–67^a^	117
10	CB7/AO	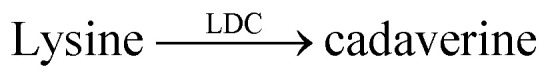	Organophosphate esters	1.3–9.1^b^	118
11	CB7/AO	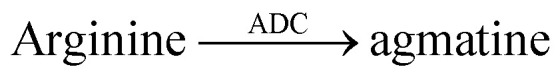	Methylmercury chloride	8 × 10^−3^	71
			Ethylmercury chloride	70 × 10^−3^	71
			Phenylmercury chloride	145 × 10^−3^	71
			HgCl_2_	365 × 10^−3^	71
			Difluoromethyl arginine	500	71
12	CB6/DSMI	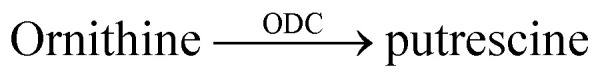	Difluoromethylornithine (DFMO)	4.4 ± 0.2^c^	60
			Epigallocatechin gallate	166 ± 8	60
	CX4/LCG	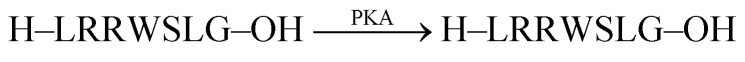	*N*-Ethylmaleimide (NEM)	n.d.^d^	109
	CX4/OX1	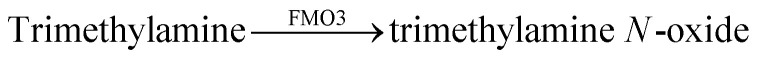	Naringin, paeoniflorin, β-ecdysterone, 18β-glycyrrhizic acid, amygdalin, albiflorin, saikosaponin A	n.d.^e^	77
	CB7/AO	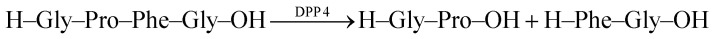	Saxagliptin	14.4 × 10^−3^	102
			Salvianolic acid C	2.7^f^	102
			Herbacetin	9.7^f^	102
			Ellagic acid	38.2^f^	102

a Thirteen perfluorinated carboxylic acids (PFCAs) and three perfluorinated sulfonic acids (PFSAs) were found to have an inhibitory effect on LDC.

b Twelve different organophosphate esters were found to have an inhibitory effect on LDC activity, among which aromatic or chlorinated alkyl groups had the inhibitory effect on LDC.

c Apparent *K*_i_ after short (<20 min) incubation times. DMFO is a suicide inhibitor, which could be also confirmed by the supramolecular tandem assay.

d NEM is a suicide inhibitor, such that the *K*_i_ was not determined.

e The inhibitors were identified by screening a compound library from a traditional Chinese medicine composed of eleven herbs with known antithrombotic activity. IC_50_ or *K*_i_ values were not determined.

f Identified from a library containing >300 compounds from Chinese traditional medicine.

Within these reports (Table 1, entries 1–8), inhibition was quantified by IC_50_ values, which is the half maximal inhibitory concentration, at which the enzymatic reaction rate is decreased by 50%. Therefore, the time-dependent fluorescence changes are recorded at varying inhibitor concentrations and the initial parts are linearly fitted to obtain enzymatic reaction rates in relative fluorescence intensity units. This is then plotted against the inhibitor concentrations and the resulting dose–response curves are analysed with the Hill equation to afford the IC_50_ values, which can be converted into inhibition constants, *K*_i_, by considering the enzyme concentration, [E]: IC_50_ = *K*_i_ + 1/2[E].^116^ This is a suitable approach that can be widely used for substrate-selective assays or when the binding constants of the products in product-selective assays are too low to afford a quantitative displacement of the fluorescent dye.

Even more desirable are, however, reporter pairs that respond linearly to the product concentration. This enables very robust assays, a straightforward quantitative evaluation, and it affords enzymatic reaction rates in molar instead of relative fluorescence units. Critical to the further development of applications of supramolecular tandem enzyme assays are thus reporter pairs with improved properties. As an example, amino acid decarboxylases were initially monitored with the CB7/dapoxyl and CX4/DBO reporter pairs.^59^ The resulting assays were sufficiently robust to enable enzyme activity detection with crude cell extracts and the determination of the Michaelis–Menten constant, *K*_m_. However, the catalytic turnover number, *k*_cat_ was not reported, because a kinetic analysis would have become too involved.^26,59^ These assays were subsequently used by Wang *et al.* to identify new inhibitors of lysine decarboxylase^117,118^ and arginine decarboxylase (Table 1, entries 9–11).^71^

**Fig. 32:**
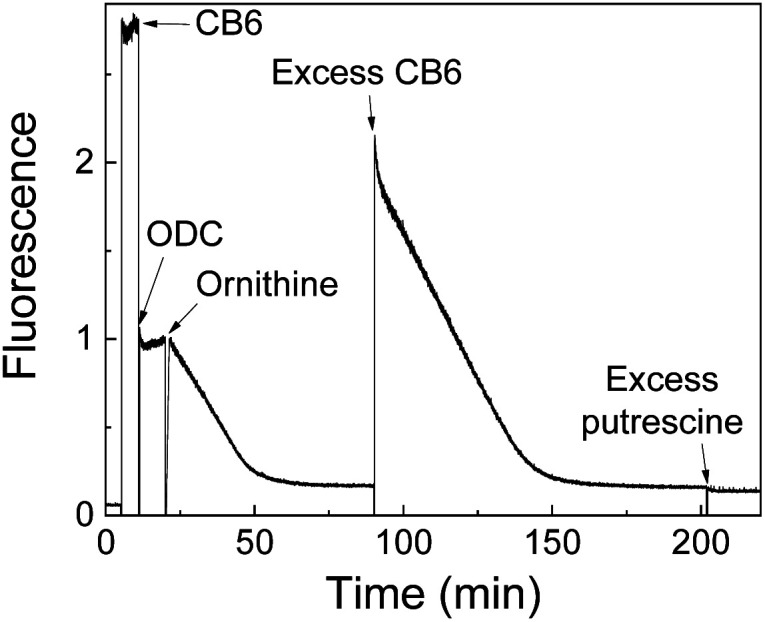
Continuous fluorescence enzyme assay for ODC. Upon addition of CB6 fluorescence is increased. Subsequently addition of ornithine, resulted in time dependent fluorescence decreased. At later stage excess of CB6 and putrescine are added for control experiments.

**Fig. 33:**
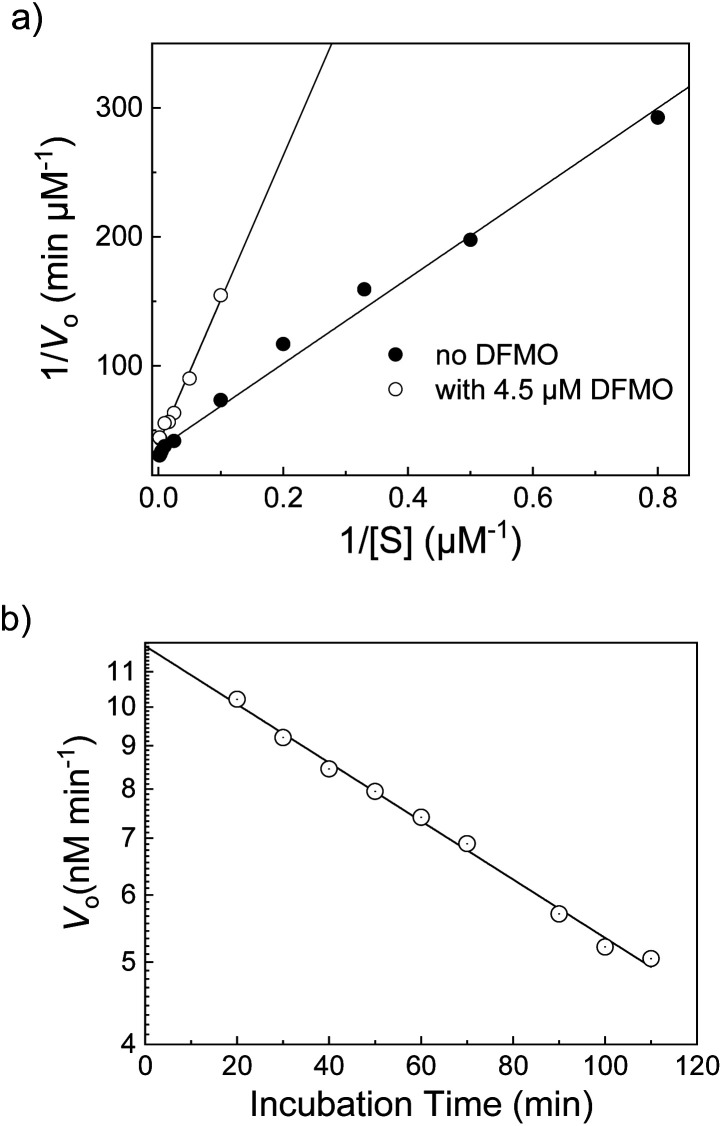
Determination of the inhibition mode of ODC by DFMO. (a) Lineweaver–Burk plot at short incubation times with DFMO (<20 min) indicating competitive inhibition. (b) Decreasing enzymatic reaction rates with increasing incubation times indicated a suicide inhibition mode of DFMO in accordance with the literature.

A further improved version of a supramolecular tandem enzyme assay for ornithine decarboxylase (ODC) was reported more recently by us.^60^ The major improvement was achieved by using DSMI as the fluorescent dye and CB6 as the macrocyclic receptor. CB6 has a higher affinity to the ODC product putrescine than CB7 or CX4 and the fluorescence of DSMI is significantly enhanced by CB6 by a factor of >100.^119^ Consequently, addition of ornithine resulted in a linear time-dependent decrease in fluorescence until a plateau region is reached (Fig. 32). In contrast to common fluorescence progress curves in enzyme assays, the plateau does not indicate that all substrate is converted into the product, but that all CB6 is occupied by the enzymatic product putrescine. This was confirmed by addition of excess CB6, which binds to the DSMI in free solution leading to a sharp increase and a subsequent time-dependent linear decrease indicative of the continued progress of the enzymatic reaction. The fluorescence units are then easily converted into product concentrations, [P], because [P] = 0 before addition of enzyme and [P] = [CB6]_tot_ = [DSMI]_displaced_ at the plateau (Fig. 32). This enabled a thorough enzyme kinetic investigation yielding a catalytic turnover number of *k*_cat_ = (0.12 ± 0.01) s^−1^ and a Michaelis–Menten constant of *K*_M_ = (24 ± 1) μM for human ODC expressed in HEK cells. Moreover, the CB6/DSMI reporter pair enabled to determine the inhibition constants of the ODC inhibitors epigallocatechin gallate (EGCG) and α-difluoromethylornithine (DFMO). The latter was selected for a more detailed investigation on the inhibition mode (Fig. 33). At short incubation times, DFMO showed competitive inhibition in a Lineweaver–Burk plot (Fig. 33a). When the logarithmic initial reaction rates at constant DFMO concentrations were plotted against varying incubation times, the expected linear dependence was found in accordance with a suicide inhibition mode of DFMO (Fig. 33b). Similar studies to identify the mode of inhibition were subsequently performed with the reporter pair CX4/LCG and PKA (see Section 5.3), which identified *N*-ethylmaleimide (NEM) as a suicide inhibitor of PKA (Table 1).^109^

Actual screening applications are commonly performed in 96 and 384-well microtiter plates and require robust assay procedures.^60,65,90,102,108,110^ The ODC assay was found to be tolerant to the required buffer additives dithiothreitol (DTT), ethylenediaminetetraacetic acid (EDTA), pyridoxal-5′-phosphate (PLP), Tween 80, and 1% DMSO and could be miniaturized to the microplate format to demonstrate its potential in HTS applications. The performance of the assay was excellent and gave a *Z*′ factor 0.96 in 96-well microplates and of 0.90 in 384 well-microplates.

Supramolecular tandem enzyme assays may thus provide an exceptional and versatile platform for rapid screening of pharmaceuticals, bioengineered enzymes, and related analytes. This was subsequently also shown by Guo and Wang, who screened a compound library with the CX4/OX1 reporter pair to identify FMO3 inhibitors (see Section 4.3 for the assay). The library contained the ingredients from a traditional Chinese medicine composed of eleven herbs with known antithrombotic activity and the screening identified several previously unknown inhibitors of FMO3 (see Table 1).^77^ Moreover, the DPP4 assay by Wang and Bi (see Section 5.1) was validated with saxagliptin as a known inhibitor and then used to screen a library of >300 compounds from traditional Chinese medicine for potential inhibitory effects on DPP4.^102^ This identified the polyphenols salvianolic acid C, herbacetin, and ellagic acid as previously unknown DPP4 inhibitors with micromolar IC_50_ values.

### 6.2 Biomarker diagnostics

The activity of specific enzymes is also an important parameter in clinical diagnostics.^120^ For example, pepsin activity is absent in saliva of healthy people, but found in patients suffering from gastroesophageal reflux disease (GERD). Pepsin activity may be even detected before patients develop any symptoms or before any diagnostic features in medical imaging such that pepsin activity in saliva is an ideal biomarker for GERD. The pepsin assay from the Guo group (Section 5.1) based on the CX4/LCG reporter pair could detect pepsin in acidic artificial saliva with an excellent limit of detection (LOD) of (329 ± 10) ng mL^−1^, which enables the prompt detection of typical pepsin levels in GERD patients.^73^

As another example, DPP4 activity was found to be higher in mice fed with a high fat diet (HFD) compared to normal fed mice and blood samples from type II diabetes patients had also a higher DPP4 activity than healthy individuals as controls. DPP4 activity is thus of interest in clinical diagnosis and the supramolecular tandem DPP4 assay (Section 5.1) was compared to a commercial ELISA kit for DPP4 quantification.^102^ DPP4 concentrations were quantified in clinical blood plasma samples based on the linear dependence of the hydrolysis rate of the Gly–Pro–Phe–Gly substrate by DPP4 in the tandem enzyme assay. The detected DPP4 concentration correlated well with the results from the ELISA kit and a higher DPP4 activity in blood plasma samples from type II diabetes patients was clearly confirmed. This example further demonstrated that supramolecular tandem assays may become a complementary tool to established immunoassays for clinical biomarker detection.

### 6.3 Screening of microbial metabolic activity

In addition to detecting enzyme activity in clinical samples, supramolecular tandem enzyme assays can also be used to explore microbial metabolic pathways. This application was inspired by the discovery that CB7 and CB8 can bind a great variety of steroids with partly astonishing selectivity.^65^ For example, CB8 binds the male sex hormone and anabolic steroid testosterone with nanomolar affinity, which can be detected by a ternary complex of CB8 with two encapsulated berberine (BE) molecules as a self-assembled fluorescent chemosensor (see Fig. 10 and 11 for structures).^121^ When the berberine molecules are displaced by testosterone, fluorescence decreases, because berberine is nearly nonfluorescent in water.^65,122^

**Fig. 34:**
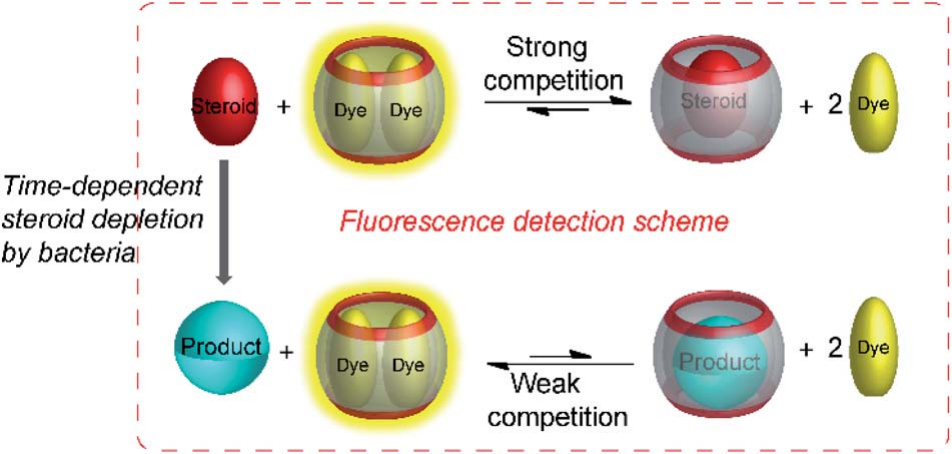
Monitoring the degradation of steroids by steroid-depleting bacteria. The steroids bind sufficiently strong to CB8 to displace the dye BE. The degradation products have a lower affinity and the CB8/BE_2_ complex can re-form as indicated by an increased intensity. The assay was conducted directly in aliquots from the bacterial culture broths.

There is an increasing interest to identify steroid-degrading bacteria for wastewater treatment, which suggested to use a supramolecular tandem assay for monitoring the depletion of testosterone by steroid-degrading bacteria (Fig. 34).^122^ Therefore, two bacterial strains were selected, *Buttiauxella* sp. S19-1 and *Marinobacter adhaerens* HP15, which are known to degrade testosterone and have no steroid-degrading capability, respectively. The experimental design involved positive and negative controls and clearly identified the testosterone-degrading capability of *Buttiauxella* sp. S19-1. The degradation was followed by a time-dependent increase in fluorescence intensity, because the degradation of the strong binder testosterone allowed the reformation of the fluorescent CB8/BE_2_ complex. Noteworthy, the assay was suitable to follow the steroid degradation down to submicromolar concentrations of testosterone in the bacterial culture media with excellent confidence intervals as assessed by a *Z*′ factor in the range of 0.52 to 0.74.

### 6.4 Optical biosensor arrays based on tandem assays

**Fig. 35:**
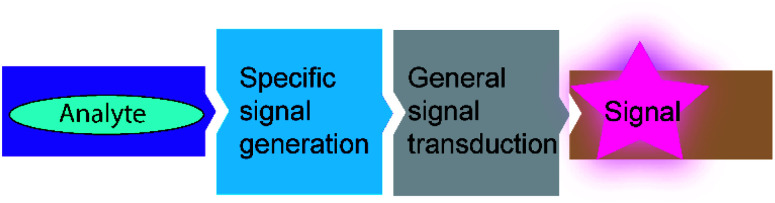
Principle of a biosensor. Typically a highly selective enzymatic reaction (*e.g.* a oxidoreductase) is used as a specific signal generator that is coupled with a signal transducer (*e.g.* a electrochemical read-out) to afford a readable signal in presence of the analyte. In the supramolecular variant, the specific signal generator is also an enzyme, whereas the transducer is a host–dye reporter pair affording an optical read-out signal.

In the applications summarized in the preceding sections, the supramolecular sensor was applied to detect variations in the activity of enzymes, *e.g.* by inhibition, in conjunction with a disease, or of different microorganisms. Alternatively, a combination of enzyme and a supramolecular sensor may afford a biosensor for the detection of the enzyme substrate as an analyte. Classical biosensors comprise an enzyme as a specific signal generator and a general transducer that converts the enzymatic reaction into a readable output signal (Fig. 35). The most established biosensors are based on electrochemical transducers coupled to oxidoreductase enzymes such as in blood glucose sensors for diabetes patients, whereas optical signal transducers have been also applied.^123,124^

The comparably low selectivity and cross-reactivity of supramolecular host–guest recognition appears ideal to apply them as signal transducers for optical sensing. One possibility to take advantage of the low selectivity and cross-reactivity is the construction of differential sensor arrays, in which several supramolecular sensors react differently towards a certain analyte to generate a pattern of sensor responses.^65,125–127^ Multivariate data analysis then allows to relate the response pattern to the presence of a certain analyte. This is reminiscent of the sensation of smell, in which a response pattern from hundreds of olfactory receptors is assigned by the brain to sensation of a certain scent. Another possibility arising from the comparably low selectivity of host–guest recognition is their use as optical signal transducers for a variety of related enzymes. The latter than function as the a specific signal generator due to the high selectivity of the enzymes.

Highly selective sensing of various amino acids has been demonstrated with amino acid decarboxylases and the CB7/dapoxyl reporter pair as a signal transducer.^90^ Four sensors were included for the detection of lysine, arginine, tyrosine, and histidine with their respective decarboxylases. The detection limit of all four amino acids was in the low micromolar range with no further optimization and no cross-reactivity between the different amino acids was noted. Moreover, the stereospecificity of the amino acid decarboxylases was exploited to assess the enantiomeric excess (ee) of d-and l-amino acids by the biosensors, which enabled the detection of the enantiomeric excess of d-amino acids up to an ee of 99.98%.

Biosensors constructed from enzymes coupled with supramolecular sensors may also be very useful for the detection of analytes in complex matrices such as body fluids or food samples. The reason is that any competing analytes in the complex matrix will afford a static and immediate response, which is constant throughout the measurement, which can be easily discriminated from the time-dependent change arising from the conversion of the analyte by the enzyme. Such endeavours are, to the best of our knowledge, so far unexplored with supramolecular tandem assays.^113,123,128^

### 6.5 Supramolecular MRI biosensing of enzyme activity

*In vivo* imaging of enzyme activity, either by optical probes or by magnetic resonance imaging (MRI) as an imaging modality, is another application area, in which supramolecular tandem enzyme assays could make an impact. Compared to passive targeting probes, enzyme-activatable imaging probes are usually most desirable, because they offer an improved signal-to-background ratio and, thus, contrast enhancement.^129,130^

One possibility to transfer supramolecular tandem enzyme assays to imaging is ^129^Xe MRI. Using hyperpolarized ^129^Xe in conjunction with chemical exchange saturation transfer (hyper-CEST) affords highly sensitive detection and has the potential to become a clinical molecular imaging modality.^131–134^ Hyper-CEST requires a sensing unit that binds Xe leading a complexation-induced shift of the ^129^Xe NMR peak and sufficiently rapid exchange with a pool of free Xe atoms. Since numerous supramolecular host molecules including cyclodextrins,^135,136^ hemicarcerands,^137,138^ calixarenes,^139–142^ cryptophanes,^143–151^ cucurbiturils,^152–159^ and cavitands^160,161^ are known to bind Xe, competitive occupation of the cavity by a substrate or product of an enzymatic reaction could modulate the Xe exchange, which becomes detectable by hyper-CEST.

**Fig. 36:**
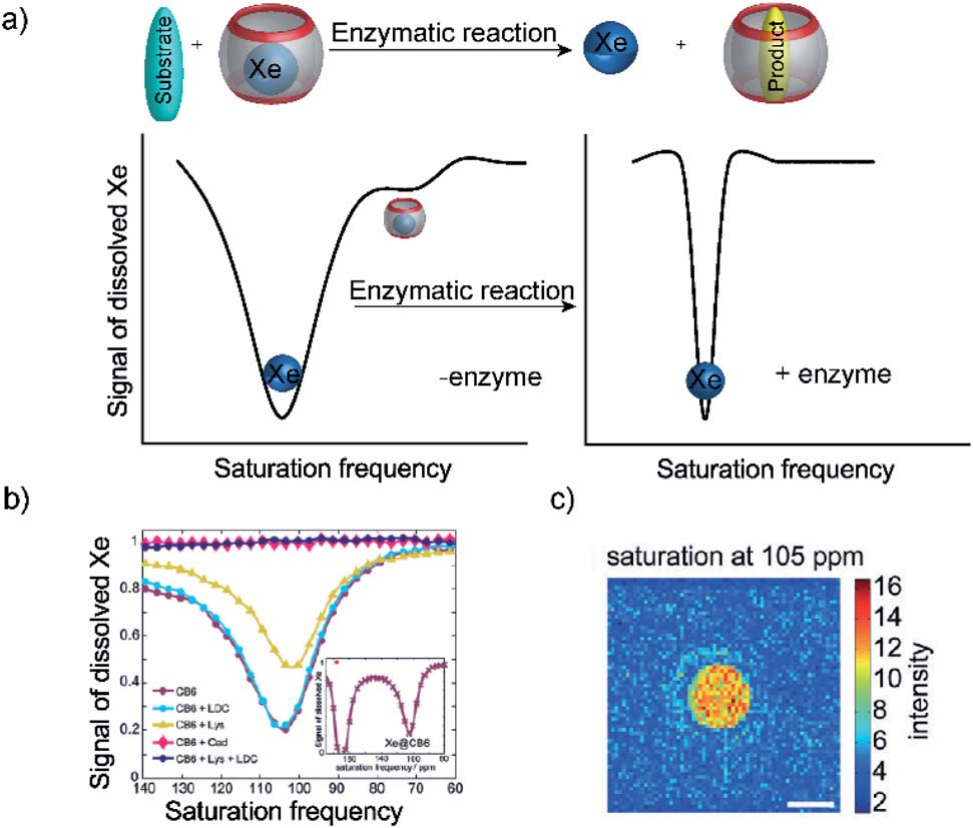
Monitoring of LDC activity based on hyperpolarized ^129^Xe biosensors. (a) Schematic diagram of change of saturation frequency before and after the displacement of Xe. (b) Change of saturation frequency during the enzymatic conversion. (c) Hyper-CEST MRI image of a phantom of two NMR tubes put into each other, where the inner compartment shows LDC activity.

Among the available Xe-binding host molecules, cryptophanes and cucurbiturils appear most promising,^131,132,134,154,155^ whereas the cavity of the former is too small to allow competitive complexation by larger guests. The application of supramolecular tandem enzyme assay in MRI with cucurbiturils follows a similar scheme as for optical detection (Fig. 36).^162,163^ When the host cavity is occupied by either the substrate or the product of the enzymatic reaction guest exchange with Xe cannot proceed leading to a decrease of the hyper-CEST signal. This was shown with lysine decarboxylase (LDC), which transforms weakly binding lysine into strongly binding cadaverine and CB6 and CB7 as potential contrast agents.^162^ With CB6, the Hyper-CEST spectra showed a resonance at *δ* = 193 ppm attributed Xe dissolved in the buffer and a resonance at *δ* = 105 ppm assigned to Xe bound to CB6. During the progress of the enzymatic reaction, the signal at 105 ppm disappeared due to occupation of the CB6 cavity by the reaction product cadaverine, which prevented CEST of Xe with CB6. When the method was transferred from buffer solution to cell lysates, no distinct resonance was observed at *δ* = 105 ppm, but the CEST response at *δ* = 193 ppm was considerably broadened. This was assigned to additional interactions of CB6 with other competitive guest in the cell lysate. The broadened signal, however, suggested to explore hyperpolarized ^129^Xe magnetization transfer with CB7 as an alternative MRI detection method, which gave an excellent contrast with phantom samples in MRI. In a subsequent extension of this work,^163^ time-resolved monitoring was achieved, which afforded initial reaction rates and product concentrations.

## 7. Conclusions

In this review, we have comprehensively summarized the current state-of-the-art of enzyme assays based on supramolecular chemosensors. Chemosensors that can discriminate enzymatic substrate and product provide a unique and innovative approach to monitor enzyme activity that is label-free, provides high sensitivity at low cost, and is compatible with HTS applications. We have summarized the design tactics for supramolecular tandem enzyme assays, that have been utilized to develop substrate-selective and product-selective tandem assays for various enzymatic reactions. This includes the initial use of low-molecular weight substrates and products that are nearly completely immersed in the cavity of the chemosensors, as well as more recent extensions to peptides that are hydrolysed or functionalized at specific amino acid side chain residues by oxidation, phosphorylation and dephosphorylation, or methylation and demethylation by different enzymes. With improved host–dye reporter pairs, the robustness of the assays was also improved leading to the first demonstration of advanced applications of tandem assays in inhibitor screening, biomarker detection, optical biosensors arrays, and MRI biosensing.

## Conflicts of interest

There are no conflicts to declare.

## Supplementary Material
